# CO-produced Nature-based Intervention Feasibility for children with ADHD Study: The CONIFAS2 study protocol

**DOI:** 10.1371/journal.pone.0359206

**Published:** 2026-10-07

**Authors:** Kathryn L. Date, Judith Cohen, Peter A. Coventry, Tracey Dorey, Natasha Green, Susan Griffin, Chao Huang, Ellen N. Kingsley, Michelle Marlow, Sarah Sumpter, Maureen Twiddy, Piran C. L. White, Kat Woolley, Hannah A. Armitt

**Affiliations:** 1 Hull Health Trials Unit, University of Hull, Hull, United Kingdom; 2 School of Nursing and Public Health, Manchester Metropolitan University, Manchester, United Kingdom; 3 Research and Development Department, Humber Teaching NHS Foundation Trust, Hull, United Kingdom; 4 Patient and Public Involvement Lead, Northampton, United Kingdom; 5 Centre for Health Economics, University of York, York, United Kingdom; 6 Institute for Clinical and Applied Health Research, University of Hull, Hull, United Kingdom; 7 Child Oriented Mental Health Innovation Collaborative, Leeds and York Partnership NHS Foundation Trust, York, United Kingdom; 8 KIDS Yorkshire and the Humber, Hull, United Kingdom; 9 Department of Environment and Geography, University of York, York, United Kingdom; 10 Public Contributor, Huddersfield, United Kingdom; 11 Smart Data Donation Service, University of York, York, United Kingdom; PLOS: Public Library of Science, UNITED KINGDOM OF GREAT BRITAIN AND NORTHERN IRELAND

## Abstract

**Introduction:**

Children with ADHD face significant unmet needs for timely post-diagnostic support in UK NHS services. Nature-based interventions represent a promising under-researched adjunct to conventional treatment. The CONIFAS2 study will assess the feasibility and acceptability of a co-produced, home-based, nature-based intervention embedded in NHS neurodiversity services.

**Methods and analysis:**

This is a feasibility randomised controlled trial assessing recruitment, retention, intervention fidelity, data collection and acceptability of both the intervention and trial design. Participants will be children diagnosed with ADHD and their parent/guardian recruited as a dyad from NHS neurodiversity services. Following baseline assessment, dyads will be randomised to either intervention, which consists of structured daily nature-based activities with psychoeducational support, delivered alongside treatment as usual, or treatment as usual only. A mixed methods process evaluation, will refine a programme theory and identify barriers and facilitators to intervention delivery, engagement and implementation in a future definitive trial. A feasibility health economic evaluation will assess the feasibility of collecting resource use, cost and health-related quality of life data to inform a future cost-effectiveness analysis.

**Dissemination:**

Findings will inform a future definitive trial and be disseminated via peer-reviewed publications, conference presentations, and community partners.

**Trial registration:**

ISRCTN ISRCTN15490570

## Introduction

### Background

Attention Deficit Hyperactivity Disorder (ADHD) is associated with higher rates of diagnosis in areas of socioeconomic deprivation, and diagnosis rates are increasing without an equivalent increase in specialist support services [1]. ADHD impacts on daily functioning and outcomes in multiple domains of health [2]. It affects 5% of UK children, with families having little access to appropriate, timely interventions post-diagnosis [3]. Recent medication shortages have heightened pressure on healthcare systems by increasing demand for alternative support options [4]. The societal impact of ADHD is significant, with the average annual cost of ADHD per child in Europe between €5,733 and €14,482 (2012 prices) [5]. A recent review highlighted the need to advance development, evaluation, implementation and dissemination of evidence-based treatment strategies to support ADHD-related difficulties [6]. Mental health issues in children and young people, especially those with ADHD, are a growing public health concern. In 2023−24, 958,200 children and young people in England had an active referral to Children and Young People’s Mental Health Services (CYPMHS) (an increase of almost 10,000 since the previous year) [7], with approximately 320,000 still waiting for treatment to begin at the end of the year (an increase of almost 50,000 on the previous year). Children with ADHD are a particularly vulnerable population with increased rates of mental health diagnosis and need [1].

Nature-based interventions represent a promising but as yet under-researched approach to support children with ADHD in managing symptoms and enhancing their wellbeing [8]. Evidence shows that contact with green space can benefit children’s physical activity, cognition, behaviour and mental health [9], with even brief exposure, such as 10 minutes outdoors, improving well-being, and optimal benefits observed at 200–300 minutes per week [10,11]. Empowering parents and guardians to develop skills and agency to support their children is an important focus of post-diagnostic ADHD provision, particularly for primary age children where indirect means of support are indicated, for example parental support and consultation [12]. Given the emphasis on empowering parents and guardians, providing support to facilitate and engage children’s contact with green space represents a potentially accessible and appropriate means to translate this evidence into everyday practice. There is however little known about how this support is enacted, experienced and whether it is feasible and acceptable as an adjunct to usual ADHD care at a time when many families experience limited access to timely post-diagnostic support and treatment options [13,14]. Furthermore, despite growing interest in nature-based interventions, little is known about how such approaches may exert their effects in children with ADHD or the mechanisms through which they may influence outcomes. This study therefore adopts a theory-informed approach, drawing on established behaviour change theory to explore plausible mechanisms of action, linking intervention components to behaviour change and wellbeing outcomes. Understanding these mechanisms will help refine the intervention and inform the design and evaluation of a future definitive trial.

### Rationale

This study builds on the CONIFAS1 study (NIHR203043), which successfully co-produced a nature-based intervention for children with ADHD with preliminary acceptability with parents and carers [15]. The co-produced intervention, termed “nature activity box”, is family-led and home-based. It aims to support the well-being and symptom reduction of children with ADHD by providing a nature-based approach in addition to existing parent-focused interventions, encouraging at least 10 minutes of daily nature engagement through structured activities and psychoeducation. Whilst the intervention is family-led and undertaken in the home or local community, it is introduced and supported by NHS neurodiversity clinicians through psychoeducation and structured follow-up support. This study seeks to explore the feasibility of integrating this intervention into NHS children’s neurodiversity services.

This research is crucial as it aims to address the significant unmet needs of children with neurodiverse conditions, particularly those with ADHD, who often struggle to access post-diagnostic support in NHS services. This need is further underscored by national gaps in ADHD service provision. According to an unpublished internal clinical service dataset on post-diagnostic waiting list numbers from Hull and East Yorkshire Children’s Neurodiversity Service, over 500 children are awaiting post-diagnostic support in the Hull and East Yorkshire region alone. The study also addresses a recognised need to reduce inequalities in access, engagement, retention, and outcomes of child and family interventions, as highlighted by the NIHR [16].

By evaluating the feasibility of the intervention in a real-world context, this research will provide important data to inform future larger-scale evaluations. Furthermore, by collecting health economic data, the study aims to assess the potential social and economic impact of the intervention on ADHD treatment.

## Materials and methods

### Aims and objectives

The CONIFAS2 study aims to assess if the delivery of the intervention is feasible and acceptable to children with ADHD, their families, and NHS neurodiversity professionals, and to evaluate the feasibility of conducting a randomised controlled trial comparing the intervention with treatment as usual.

The study objectives are:

- To determine the feasibility and acceptability of key trial processes, including recruitment, retention, randomisation and collection of health/wellbeing, process and health economic outcome data;
- To explore the feasibility and acceptability of delivering and receiving the intervention within NHS neurodiversity services from the perspectives of children, families and NHS staff;
- To examine how the intervention is implemented in practice, including how contextual factors influence delivery and outcomes;
- To collect quantitative and qualitative data to refine the programme theory, informing how and why the intervention may work.

Together these findings are designed to inform the design of a future large-scale effectiveness trial.

### Study design

This is a two-arm feasibility randomised controlled trial with embedded mixed-methods process evaluation and feasibility health economics evaluation. The study is being conducted across children’s neurodiversity ADHD services, initially within Humber Teaching NHS Foundation Trust (HTFT) and Navigo Community Interest Company (CiC) who hold the NHS contract for ADHD service delivery in North East Lincolnshire, with phased inclusion of Cornwall Partnership NHS Foundation Trust and Leeds Community Healthcare NHS Trust to support recruitment. The study will compare the intervention plus treatment as usual (TAU) to TAU alone when provided to families on the post-diagnosis ADHD support pathway. The study will assess trial and intervention feasibility over a 6-week intervention and follow-up period. The protocol is reported in accordance with the SPIRIT 2025 reporting guidelines [17]. The SPIRIT schedule of enrolment, interventions and assessments is shown in Fig 1 and the completed SPIRIT checklist can be found in the Supplementary Information (S1 Checklist).

**Fig 1:**
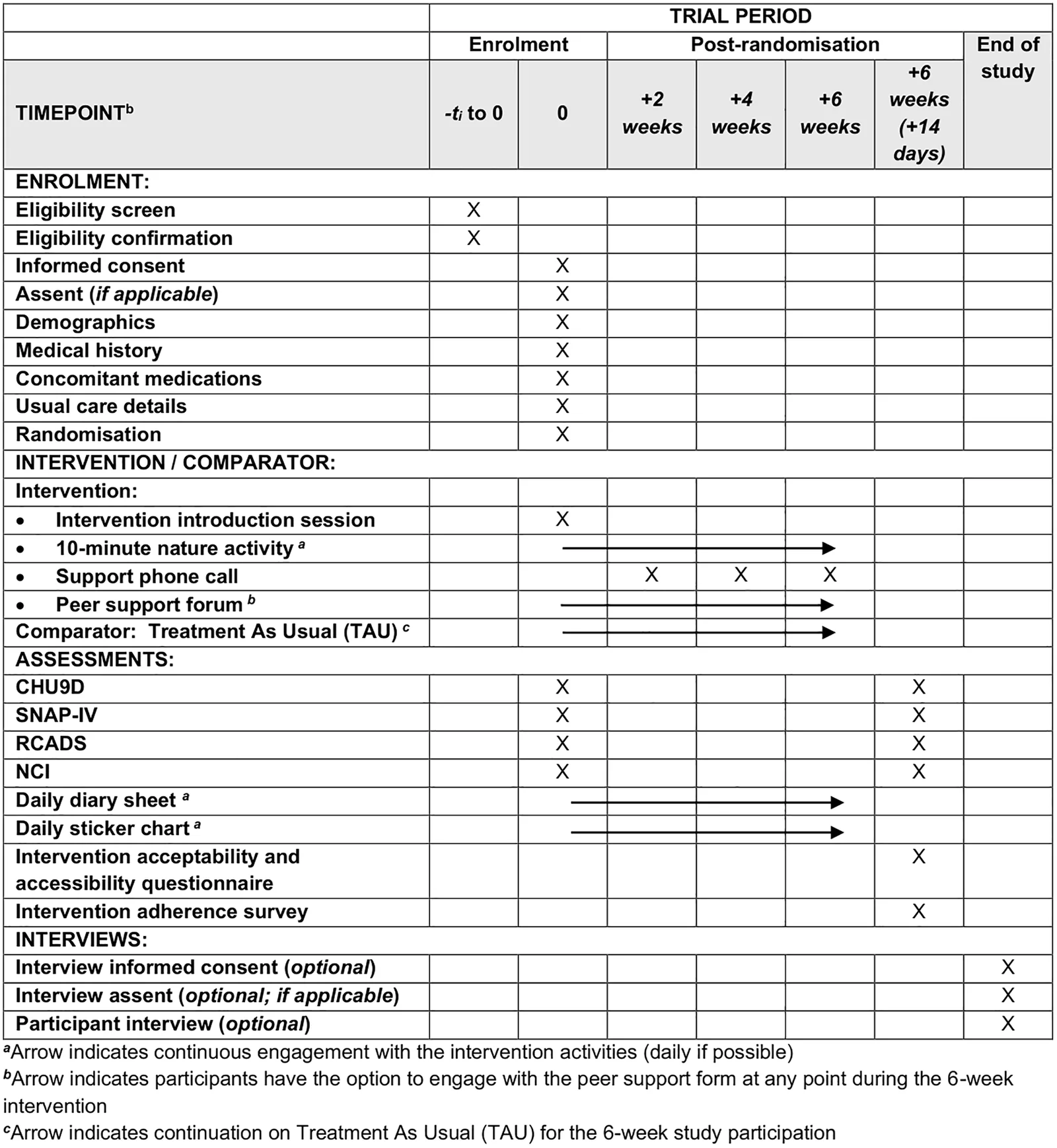
SPIRIT schedule of enrolment, interventions and assessments.

### Eligibility criteria

Eligibility to participate in the trial will be initially assessed by the site Principal Investigator (PI) or delegated member of the site research team. The CONIFAS2 central research team will then confirm eligibility at first contact with the participant.

The study aims to recruit children aged 6–11 years diagnosed with ADHD who have been referred to or are currently under the care of NHS neurodiversity services. Children aged 6–11 years have been selected to reflect the primary school age group for whom parent‑mediated interventions are most appropriate and with whom the intervention was co-produced. For each child participant, a parent/guardian will also be recruited for completion of outcome measures. Both the child and their parent/guardian must have sufficient English comprehension (equivalent to CEFR B1 or above) to participate in the study.

Additional intervention development work in a separate study work package will investigate cultural sensitivity, translation and inclusivity in order to support broader inclusion in a future full-scale trial.

Children will be excluded from participating in the study if they are considered to pose a risk of harm to themself or others whilst under parental supervision, or to have profound additional difficulties that would hinder participation in nature-based activities as determined by their parent/guardian.

### Sample size

As a feasibility trial, the study is not powered to formally detect the clinical effectiveness of the intervention versus TAU. The sample size was calculated to ensure reliable estimation of key feasibility parameters, detection of signal of efficacy, and to provide variance estimates to inform the sample size calculation for the future full-scale trial.

The proposed sample size is 84 participants (42 per arm). This allows for 35 evaluable participants per group, consistent with recommendations for external pilot/feasibility study precision [18], whilst accounting for an anticipated 15% attrition rate.

### Recruitment

Initial identification and screening of potentially eligible participants will be undertaken by the site clinical teams, who will contact the parent/guardian and provide an easy-read pictorial-based summary information sheet to introduce the study. The easy read information sheet is designed to support participation among parents/guardians with low literacy, comprehension difficulties, or language processing needs.

Following confirmation of eligibility, verbal consent will be sought from interested families to share contact information with the CONIFAS2 central research team. The research team will then provide each family with two Participant Information Sheets (PIS) – one for parents/guardians and an easy read version for the child. A video version of the study information has also been created to support inclusion of parents/guardians with poor literacy skills or comprehension difficulties.

### Informed consent

Informed consent will be received by appropriately trained and delegated members of the research team prior to the participant taking part in any study-related activities. Consent will primarily be completed using remote electronic consent (e-Consent) via Box Sign eSignature platform or remotely witnessed verbal consent. This will mainly be obtained during a telephone or video call, although a face-to-face visit can be arranged if requested.

Parents/guardians will be asked to provide consent for both their own and their child’s participation in the study. Child participants will also be invited to complete a simple assent form (if appropriate) to indicate their willingness to participate. Participants who view the video PIS will provide consent via remote witnessed consent, using a simplified version of the consent form. The consent form includes a statement to make it clear that anonymised study data may be used and shared to support future ethically approved studies.

Enrolment of consenting participants in the study database will automatically generate a unique pseudonymised Participant ID Number, which will be recorded during the consent process to complete the consent form. Copies of all completed consent and assent forms will be uploaded into the study database to facilitate remote central monitoring. All consent procedures will be undertaken in accordance with Good Clinical Practice and local governance policies.

### Randomisation

Following completion of baseline outcome measures, all enrolled participants will be randomised sequentially using a purpose-built web-based data capture system with integrated randomisation function hosted by Hull Health Trials Unit (HHTU) within REDCap Cloud (RCC).

Participants will be randomised in a 1:1 ratio to either the intervention or TAU. The allocation will use random permuted blocks and be stratified by site. The sequence will be generated by an independent statistician and will remain concealed from study staff. An unblinded member of the central research team will receive randomisation alerts containing the unblinded allocation details. They will inform clinical sites and participants of the allocation and arrange delivery of intervention boxes accordingly. Allocation concealment will be maintained through the secure, automated randomisation function within RCC, preventing study staff from predicting or influencing assignment.

### Blinding

Members of the research team collecting outcome measure data will remain blinded to group allocation where possible. Baseline data will be collected prior to randomisation, and standardised language will be used during follow-up calls with families to try to prevent unintentional unblinding. Detailed information relating to serious adverse events (SAEs) will also remain concealed from blinded researchers.

In order to determine whether completion of intervention-specific questionnaires should be requested during the follow-up call, these events will remain hidden in the study database until researchers are unblinded once all other outcome measures have been completed.

The extent to which blinding can be maintained within the study will contribute to the assessment of study feasibility. Due to the nature of the intervention, participants and families cannot be blinded to group allocation.

### Intervention

The intervention is a multi-component, home-based, co-produced, nature-based activity intervention for children with ADHD and their families. It consists of a nature-based activity box, support from a professional and optional peer support.

The nature activity box is designed to encourage families to undertake at least ten minutes of daily nature exposure. It contains activity cards and starter activities to guide engagement, psychoeducational resources, and a daily diary sheet and sticker calendar to track adherence. The ten-minute dose of nature was designed to be a practical and achievable duration based on discussions with families during CONIFAS1 co-production workshops. The intervention is designed for accessibility in both urban and rural environments, with several useful activities and suggestions for families with reduced access to green space.

A neurodiversity ADHD service clinician from a participating NHS service will provide an initial introduction to the intervention (psychoeducation and practical advice), using videos and additional guidance created by the study team, followed by fortnightly telephone support (three calls of approximately 30 minutes in total over the six-week intervention period). These calls are designed to provide encouragement, review progress, support goal-setting, and identify and address barriers to intervention engagement. To promote consistency of delivery, clinicians will follow a structured fidelity checklist outlining key topics to be covered.

Families of participants randomised to the intervention group will also have the option of joining an online community of experience forum moderated by Kids charity organisation, providing an opportunity to connect with other families taking part in the study. This element was added to the intervention in response to the feedback received from the co-production and user testing in CONIFAS1.

### Comparator

Participants in the control group will continue to receive TAU according to their local NHS neurodiversity service provision. This typically includes placement on a waiting list, periodic clinical review, and crisis management as needed, but may also include medication or signposted services (e.g., for family and education support, health and wellbeing, mental health, condition-specific help, community and activities). Details of TAU received and any changes during the study will be recorded for each participant.

At the end of the study, control group participants will receive a small nature-themed goodie bag with selected intervention box items, such as activity cards and starter items.

### Study procedures

Study participation is outlined in Figs 1 and 2. Following study enrolment, all participants will complete the same set of baseline assessments prior to randomisation. This will take place either during the consent call or on a follow-up call within 2 weeks of consent. These measures are expected to take 30–60 minutes to complete with support from the researcher where required.

**Fig 2:**
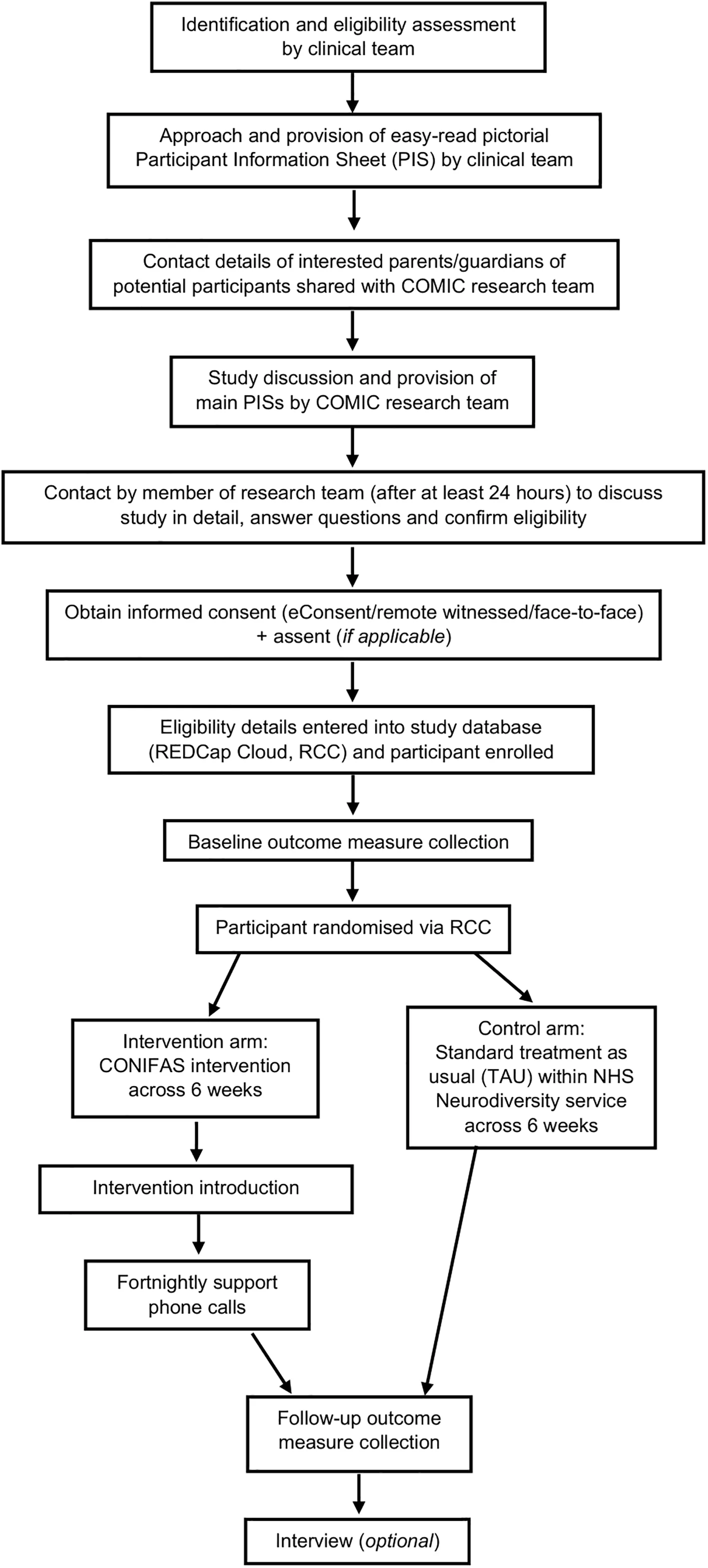
Study flow diagram.

An intervention box will be provided via post to families randomised to the intervention arm. They will then receive an intervention introduction from their clinical team and fortnightly support calls across their 6-week intervention period in addition to TAU. Families in the control arm will continue to receive TAU with no additional intervention.

All candidate outcome measures will be repeated at 6 weeks post-baseline (or within 14 days after). Additional bespoke questionnaires on language accessibility and intervention acceptability will be collected at follow-up for those in the intervention arm. A subset of participants will then also be invited to take part in qualitative interviews.

### Process evaluation

An embedded mixed-methods process evaluation will be used to further examine the feasibility, acceptability and implementation of the intervention and associated trial procedures. The process evaluation aims to:

- Assess the fidelity and quality of intervention delivery
- Identify barriers and facilitators to engagement and implementation
- Explore potential mechanisms of action underpinning the intervention
- Examine how the intervention fits within existing NHS pathways
- Inform refinement of the intervention and a future definitive trial.

The Behaviour Change Technique (BCT) Taxonomy v1 [19] will be used to identify the core components of the main intervention delivery system and proposed theory-linked BCTs. BCTs will be mapped to proposed mechanisms of action (MOA) using the Theory and Technique Tool [20] and, where necessary, the Theoretical Domains Framework (TDF) [21].

Findings on identified intervention content and MOA will be used to further refine the initial logic model developed during the CONIFAS1 study [15] and inform development of interview questions to explore the proposed BCT/MOA.

Semi-structured qualitative interviews to explore acceptability, contextual factors, intervention mechanisms and trial procedures will be conducted online/via telephone with parent/child dyads at the end of their 6-week study participation. Participants will be purposively sampled to include both those completing the intervention and non-completers, and aiming to ensure diversity in site, age, gender and comorbidities. A small sample of older children will be interviewed alone, with parental agreement. A sample size of 12–15 participant dyads (parent/child) is proposed; however, interviews will continue to be conducted until theme saturation is achieved.

Additionally, online interviews with NHS staff involved in delivery and/or recruitment (n = 4–5) will explore the delivery of the intervention and study, and discuss implementation issues. The sample will be purposively selected where possible to represent site, band and professional experience.

### Study outcomes and assessments

Feasibility outcome measure data will be collected to inform the optimum design and processes of a future randomised controlled trial (RCT). This will include recruitment and retention, data quality and integrity, acceptability and accessibility of the intervention and trial procedures, and intervention fidelity and adherence.

Recruitment rate, eligibility to consent ratio and retention rate will be measured as indicators of recruitment success, while demographic data relating to age, gender, ethnicity, socio-economic status, Special Educational Needs and Disability (SEND) status, comorbid conditions, medication use, service wait times and previous Children’s Neurodiversity Service involvement, will be collected to inform the feasibility of recruiting a diverse sample.

Acceptability and accessibility of the intervention and trial processes will be assessed using bespoke acceptability and accessibility measures as used in the CONIFAS1 study. A bespoke clinician-completed fidelity checklist will be utilised to record the content of introductory sessions and fortnightly calls as a measure of fidelity of intervention delivery by NHS staff. A daily diary sheet (parent/guardian completed) and sticker chart (child completed) will be used to record the frequency and duration of engagement with the intervention and types of activity or activities completed. An anonymous parent/guardian-completed questionnaire hosted via RCC will be used to assess intervention adherence.

The study will also monitor the collection feasibility of candidate outcome measures for the future RCT. The candidate primary outcome will be the Child Health Utility 9D (CHU9D) index, a paediatric generic preference-based measure of health-related quality of life which also supports calculation of quality adjusted life years (QALYs) for use in cost utility analysis [22–24]. This will be proxy completed for all children under 7 years and either proxy- or self-completed for children 7 years and older.

Three additional candidate secondary outcome measures will also be completed:

- The SNAP-IV 26-item scale, an abbreviated version of the Swanson, Nolan and Pelham (SNAP) questionnaire [25,26], which is a widely used rating scale for ADHD symptoms [27];
- The Revised Child Anxiety and Depression Scale – Parent Version (RCADS-P), a 47-item questionnaire with subscales to help monitor symptoms of anxiety and depression in children and young people [28];
- The Nature Connection Index (NCI), a 6-item scale used to measure nature connectedness [29].

SNAP-IV 26-item scale and RCADS-P will be completed by the parent/guardian while the NCI will be completed by the child. The parent/guardian will have the option to return the child’s response to the NCI separately via email within 24 hours if the child does not wish to be part of the call.

As part of the process evaluation, the first iteration of the programme theory will be refined utilising data collected in the qualitative interviews with children, families and NHS staff. The topic guides will be designed to further explore intervention and RCT design acceptability, focusing on experiences of engaging with the intervention as a parent/child participant as well as delivering the intervention within the NHS care pathway as NHS staff. Staff experiences of rolling out the intervention in practice, and relative barriers and enablers associated with this, will be used to guide future implementation and scale-up strategies.

### Safety considerations

Participants will be monitored for any adverse events (AEs) at key contact points throughout their participation in the study. The AE reporting period begins from the point of consent and ends 30 days after completion of follow-up outcome measures.

Parents/guardians of participants in both study arms will be provided with information relating to adverse events of special interest (AESIs) defined as any deterioration in mental health, including self-harm, self-injurious behaviour and reported suicidal ideation or actuation, and will be encouraged to report their occurrence during their study participation to the study team.

A robust serious adverse event (SAE) reporting process will be in place, with direct reporting, automatic alerts to the study team, and documentation of AE review and assessment via the study database. If an SAE is identified, the study team will follow established NHS clinical pathways to ensure appropriate and timely support.

### Data collection and management

The study database and data processes will be developed and managed by Hull Health Trials Unit (HHTU). All HHTU data systems are within scope of the HHTU NHS Data Security and Protection Toolkit. All information collected about participant dyads during the trial is confidential and will be held in accordance with GDPR 2018.

Participant data will be collected and stored in a purpose-built, secure online electronic data capture (EDC) system with granular role-based user access permissions, held within the HHTU instance of the cloud-based system, REDCap Cloud (RCC). The database will be built and validated according to study-specific requirements outlined in a Data Management Plan agreed by the CI, Sponsor, HHTU and study statistician. All activity within RCC is subject to a full user audit, with both automated and manual checks to monitor data quality and completeness.

All study participants will be automatically assigned a unique identifying number at the point of enrolment onto the study database. eConsent signatures and associated data will be captured using Box Sign cloud-based eSignature platform. Although copies of participant consent forms will be stored on Box, they will not be exported as part of the final trial dataset.

Individual participant data required by the trial protocol will be entered, as far as practicable, directly into online electronic case report forms (eCRFs) within the study database in real time. Where this is not possible, for example due to technical, connectivity or system access issues, data will be collected on paper forms and transcribed into the study database as soon as possible thereafter.

Information on any AEs, SAEs or related unexpected serious adverse events (RUSAEs) occurring during the trial will be collected in the study database and coded using the Medical Dictionary for Regulatory Activities (MedDRA) prior to database lock.

Qualitative interviews will be audio or video recorded and transcribed verbatim, and identifiable data will be redacted from transcripts and replaced with unique identifiers prior to analysis. Audio/video files and resulting anonymised transcripts will be held in the secure HHTU instance of Box.com collaborative cloud file storage system and/or securely in COMIC MS Teams on One Drive, dependent on the chosen interview platform. Original audio/video-recorded data will be destroyed once transcription is completed and allocation of unique identifiers has been assigned.

Following trial completion and resolution of all data queries, the database will be locked. The final dataset will be exported from RCC for analysis and stored in HHTU Box Instance with restricted access. A copy of the final trial dataset and notification of completion of trial participant follow-up and interviews will be sent to the Sponsor before the randomisation list will be released by HHTU prior to statistical analysis. The locked study dataset will be transferred to the trial statistician at the University of Hull and the health economist at the University of York for analysis. A copy of each individual site’s data will be sent to the site, and a copy of the final trial dataset will be archived by HHTU. Each site will receive a copy of their locked dataset before the final analysis takes place.

Analysis datasets will be transferred using HHTU Box, with a specific secured folder created. All hard copy trial documents will be retained at study sites and by the central research team in a secure location during and when the trial is finished in accordance with data protection requirements for the retention of research data and local data management policies.

Anonymised participant data will be made available upon request following consideration by the study team on a case-by-case basis. Requests should be made to the CI and will be considered by the Trial Management Group (TMG).

### Statistical analysis

The trial will be reported in accordance with the CONSORT 2010 statement extension to pilot and feasibility trials [30]. Analyses will be conducted as detailed in a Statistical Analysis Plan, which will be developed and finalised prior to database lock.

As this is a feasibility study, no formal hypothesis testing will be undertaken. Analyses will be descriptive, with continuous outcomes summarised using means and standard deviations, ordinal data using medians and interquartile ranges, and categorical data using counts and percentages.

A CONSORT flow diagram will be used to display patient recruitment and data completeness. A summary of baseline demographic and clinical characteristics will be presented by trial arm in table format to explore any between group differences.

Feasibility outcomes will be presented in a table with reference to performance against pre-specified stop-go criteria, which have been mapped to key feasibility objectives (see Table 1). The recruitment rate, consisting of the eligibility and consent rate, will be calculated with 95% confidence intervals.

**Table 1:** Study progression criteria.

Criteria	GREEN (progress to large-scale RCT)	AMBER (consider changes to intervention or trial design)	RED (do not progress – intervention delivery or RCT not feasible)
**Site set-up:** Site approvals obtained, staff trained to deliver intervention, open to recruitment	≥2 sites fully achieve criteria	1 site achieves criteria, important insights from other sites to inform how to overcome challenges for a future study	No sites meet criteria, not possible to overcome challenges
**Recruitment rate:** Aim to recruit 84 participants across sites over 10 months	Average ≥5 participants recruited per month	Average 3–4 participants recruited per month	Average <3 participants recruited per month
**Fidelity of intervention delivery:** Intro session and fortnightly calls occurred and content delivered	All core components of the intervention delivered	>75% of core components delivered	≤75% of core components delivered
**Engagement/ adherence with the intervention:** Attendance at intro session, fortnightly calls x3, completion of daily intervention tasks	Attendance at intro session and all calls, completion of >80% of intervention tasks	Attendance at intro and 2/3 calls, completion of 50–80% of intervention tasks	Attendance at intro session and 1/3 calls, completion of <50% of intervention tasks
**Acceptability of intervention/ RCT:** Qualitative process evaluation	No major concerns identified	Some acceptability issues identified that can be addressed with adaptations/ refinement to the intervention or study design	Major acceptability issues that would require substantial re-design of intervention or study
**Data collection:** Data completion rates, CHU9D (candidate primary outcome) at baseline and 6 weeks)	85-100% completion rate	60-84% completion rate	<60% completion rate

The candidate primary outcome measure for the definitive trial, CHU9D, will be summarised by allocated group at baseline and 6-week follow-up. Although not formally powered to assess effectiveness, an effect size estimate (mean difference with 95% CI) will be calculated to explore signal of efficacy. This will help inform the sample size calculation for a future trial.

Secondary outcome measures (SNAP-IV, RCADS, NCI) will be reported using descriptive statistics by trial arm at each time point.

Safety data (AEs and SAEs) will also be summarised descriptively for each trial arm.

### Qualitative data analysis

Qualitative data will be analysed in NVivo using the five iterative steps of Framework Analysis [31,32]: familiarisation, identification of a thematic framework, indexing, charting and mapping and interpretation, allowing both theory-driven and inductive coding. The analysis will focus on answering research questions on the acceptability and feasibility of delivering the intervention, as well as refine the programme theory, with the overall aim of identifying key themes, patterns and implications for future research.

### Health economic analysis

Although a formal health economic evaluation will not be conducted as part of this feasibility study, the health economic analysis is designed to assess the feasibility of collecting cost-effectiveness data to inform the development of a full cost-effectiveness analysis in a future definitive RCT.

The analysis will use data from the qualitative interviews to capture preliminary insights into family dynamics and patterns of resource use during the intervention. It will review information gathered in the daily diaries regarding activities to explore their potential impact on household finances, and costs of intervention materials will be calculated and reported. Data collected using the clinician acceptability questionnaire will be used to examine the feasibility of measuring the impacts of the intervention on clinician time, to determine an overall cost of intervention delivery. The CHU9D data will also be used to provide utility values allowing quality-adjusted life years (QALYs) to be estimated.

### Patient and public involvement and engagement (PPIE)

Study design elements and intervention components were refined in consultation with the CONIFAS1 co-production group and during two workshops held with children in local schools. Extensive consultation with service providers informed the decision to deliver the intervention in NHS children’s neurodiversity services.

A study-specific PPIE group consisting of approximately 8–10 parents and their children with an ADHD diagnosis will meet face-to-face on up to eight occasions throughout the study, with a celebration event at the end of the study. Existing networks will also be used to seek wider viewpoints from other groups of young people, for example Nothing About Us Without Us.

PPIE will be structured in accordance with the Patient Involvement in Research Impact Toolkit (PIRIT) on the NIHR Learning for Involvement platform and will develop iteratively as the study progresses. A variety of methods will be used to enable productive involvement including creative activities and online or 1:1 sessions if required. Contributions will be discussed and agreed with the group but will likely include co-creation of accessible study summaries, interview schedule and data analysis refinement, and identification of barriers to participation and possible solutions.

PPIE will be evaluated at the end of the study using a combination of informal questions and a formal tool.

### Ethical considerations

The trial protocol has been reviewed and approved by the East Midlands – Derby Research Ethics Committee (REC ref. 25/EM/0224) and the HRA. The study is registered on ISRCTN (ISRCTN15490570). The ISRCTN registry record will be updated in accordance with any modifications to the study.

### Current trial status

The study opened to recruitment on 26 January 2026 with the first participant recruited on 17 February 2026. Study recruitment is expected to close on 31 October 2026.

The current protocol version is V1.6 13.AUG.2026 (see S1 Protocol). No substantial modifications have been submitted since the original REC approved version.

### Trial oversight

The study sponsor is Humber Teaching NHS Foundation Trust. Hull Health Trials Unit (HHTU) and the Child Oriented Mental Health Innovation Collaborative (COMIC) at Leeds and York Partnership NHS Foundation Trust (LYPFT) are responsible for trial implementation and management. Central and site monitoring will be performed by HHTU in accordance with a sponsor-approved monitoring plan.

A Trial Management Group (TMG) will meet monthly for the first 2 months and thereafter every 3 months across the full study, and will be responsible for overseeing trial delivery and operations. An independent Trial Steering Committee (TSC), including independent clinical and methodological experts, an independent statistician and a PPIE representative, will meet approximately every 6 months. The TSC will provide overall trial supervision and advice on behalf of the study sponsor and funder, and is responsible for making executive decisions regarding the continuation of the trial. This will include monitoring of accruing safety data. If the risk profile changes during the study, the need for a Data Monitoring and Ethics Committee (DMEC) will be reviewed.

### Dissemination

The study will produce a range of outputs including the physical intervention and training ready for delivery, outputs to communicate the results of the study and resources to inform a future RCT. A finalised version of the nature activity box intervention element will be produced as well as an accompanying training package to support children’s neurodiversity service staff with intervention delivery

Findings will be published in peer-reviewed journals and disseminated more widely through accessible summaries (infographics, short reports and posters) on affiliated partner organisation websites, social media and presentations at conferences. Authorship will be determined according to the International Committee of Medical Journal Editors (ICMJE) guidelines. Further formats for dissemination will be decided upon by PPIE in collaboration with the research team. Findings will also be presented at a CONIFAS2 dissemination event at the end of the study.

Health economic, acceptability and feasibility data will be used to inform reports to take to a wider strategic level for engagement for a full RCT. At a service provider level, data collected on delivery acceptability and feasibility, and identified barriers, will be used in outputs to engage with other children’s neurodiversity services teams to create a list of sites for participation in a full RCT.

## Discussion

This protocol describes a feasibility RCT designed to assess the acceptability, deliverability and implementation of the intervention within NHS children’s neurodiversity services. Key strengths of the study include its co‑produced, theory‑informed intervention design and evaluation framework, the incorporation of a mixed‑methods process evaluation, and the embedding of real‑world NHS clinicians in intervention delivery. The study builds directly on the CONIFAS1 development work and draws on a preliminary logic model and behaviour change techniques to ensure a clear theoretical foundation for future evaluation. This logic model will be refined through integration of qualitative and quantitative findings from the process evaluation.

Nature-based interventions have shown promise for improving wellbeing, mental health, physical activity and cognitive outcomes in children, but evidence relating specifically to children with ADHD remains limited and there is little understanding of how such interventions can be implemented within routine healthcare settings. This study addresses these gaps by evaluating a co-produced intervention within NHS neurodiversity services while also examining potential mechanisms of action and implementation processes. By combining feasibility assessment with theory-informed process evaluation, the study seeks to contribute to the growing literature on accessible, non-pharmacological approaches to supporting children with ADHD and their families.

Several anticipated feasibility considerations may affect implementation, including variation in treatment‑as‑usual across sites, demands on families to engage in daily home‑based nature activities, and challenges in maintaining researcher blinding due to the nature of the intervention. Differences in service capacity, follow‑up burden, and families’ access to safe outdoor space may also influence both adherence and data completeness. The study design incorporates mechanisms to identify and address these issues, including monitoring of fidelity and adherence, acceptability measures, and qualitative exploration with families and NHS staff.

Findings from this feasibility study will inform refinement of the intervention content, delivery processes, and programme theory, as well as optimisation of trial procedures for a definitive evaluation. Feasibility data, including recruitment, retention, outcome completion, fidelity and early signals of efficacy, will support progression decisions and inform sample size estimation for a future RCT. Collectively, this work will provide the evidence required to design a robust, scalable evaluation of the intervention, with the ultimate aim of improving post‑diagnostic support for children with ADHD and their families within NHS neurodiversity pathways.

## Supporting information

S1 Checklist SPIRIT Checklist. (DOCX)

S1 Protocol CONIFAS2 Protocol. Current approved CONIFAS2 Protocol Version 1.6 13.AUG.2026.
(PDF)
