# Supplementary material for: CO-produced Nature-based Intervention Feasibility for children with ADHD Study: The CONIFAS2 study protocol
Source: PLoS One. 2026 Oct 7;21(10):e0359206. doi: 10.1371/journal.pone.0359206 (PMC13645079; doi:10.1371/journal.pone.0359206)
Supplement: Supplementary file 1 [file pone.0359206.s002.pdf]

CO-produced Nature-based Intervention Feasibility for children with ADHD Study

(CONIFAS2)

**Protocol Version**	V1.6 13.AUG.2026
**HHTU Reference Number**	P227
**Sponsor Reference Number**	25/07/707
**Funder Reference Number**	NIHR207869
**IRAS ID Number**	346379
**REC Reference Number**	25/EM/0224
**ISRCTN Reference Number**	ISRCTN15490570

This document describes a clinical trial and provides information about procedures for entering participants. The protocol is not intended for use as a guide to the treatment of other patients. Amendments may be necessary; these will be circulated to known participants in the trial.

SIGNATURE PAGE

The undersigned confirm that the following protocol has been agreed and accepted and that the Chief Investigator agrees to conduct the study in compliance with the approved protocol and will adhere to the principles outlined in the Declaration of Helsinki, GCP guidelines, the Sponsor's (and any other relevant) standard operating procedures (SOPs), and other regulatory requirements.

I agree to ensure that the confidential information contained in this document will not be used for any other purpose other than the evaluation or conduct of the investigation without the prior written consent of the Sponsor.

I also confirm that I will make the findings of the study publicly available through publication or other dissemination tools without any unnecessary delay and that an honest, accurate and transparent account of the study will be given; and that any discrepancies and serious breaches of GCP from the study as planned in this protocol will be explained.

**For and on behalf of the Study Sponsor:**	
Signature:	Date:
Name (*please print*):	Position:
**Chief Investigator:**	
Signature:	Date:
Name (*please print*):	

This study/project is funded by the NIHR Research for Patient Benefit research programme (NIHR). The views expressed are those of the author(s) and not necessarily those of the NIHR or the Department of Health and Social Care.

KEY CONTACTS

**Chief Investigator**	**Dr Hannah Armitt, Clinical Research Psychologist,**<br>Research and Development Department,<br>Humber Teaching NHS Foundation Trust,<br>Trust Headquarters, Willerby Hill,<br>Willerby, HU10 6ED<br>Tel: 01482 301726 Email: hannah.armitt@nhs.net
**Sponsor**	**Humber Teaching NHS Foundation Trust,**<br>Trust Headquarters, Willerby Hill,<br>Willerby, HU10 6ED
**Study Coordinators**	**Ellen Kingsley, Research Fellow and Trial Coordinator,**<br>Leeds & York Partnership NHS Foundation Trust,<br>COMIC Research, Building 17, 15 Priory Street,<br>York, YO1 6ET<br>Email: e.kingsley@nhs.net<br><br>**Dr Kathryn Date, Trial Manager,**<br>Hull Health Trials Unit,<br>3rd Floor Allam Building, University of Hull,<br>Hull, HU6 7RX<br>Email: kathryn.date@hyms.ac.uk
**Study Statistician**	**Dr Chao Huang**<br>Institute of Clinical and Applied Health Research (ICAHR),<br>3rd Floor Allam Building, University of Hull,<br>Hull, HU6 7RX<br>Email: chao.huang@hyms.ac.uk
**Funder**	Research for Patient Benefit (RfPB)<br>NIHR<br>15 Church Street,<br>Twickenham, TW1 3NL<br>Email: rfpb@nihr.ac.uk
**Key Protocol Contributors**	Dr Hannah Armitt<br>Professor Judith Cohen<br>Professor Susan Griffin<br>Dr Maureen Twiddy<br>Dr Kathryn Date<br>Dr Tracey Dorey<br>Ellen Kingsley<br>Saddaf Shaheen

i. TABLE OF CONTENTS

ii. LIST OF ABBREVIATIONS 6
iii. TRIAL SUMMARY 9
iv. STUDY FLOW CHART 11
v. SCHEDULE OF EVENTS 12
vi. TRIAL ROLES AND RESPONSIBILITIES 13
1. BACKGROUND & RATIONALE 13
1.1. Background 13
1.2. Rationale 14
2. AIMS AND OBJECTIVES 15
2.1. Trial Outcomes 15
2.1.1. Outcome Measures 16
3. TRIAL DESIGN 19
3.1. Study Design 19
3.2. Study Setting 19
4. RECRUITMENT 19
4.1. Eligibility 19
4.1.1. Inclusion Criteria - Child 19
4.1.2. Exclusion Criteria – Child 19
4.1.3. Inclusion Criteria – Parent/Guardian 20
4.1.4. Exclusion Criteria – Parent/Guardian 20
4.2. Screening 20
4.2.1. Enhancing Equality and Diversity 21
4.3. Informed Consent 21
4.4. Participants who Choose Not to Participate 22
4.5. Withdrawal of Participants 22
5. ENROLMENT AND RANDOMISATION 23
5.1. Enrolment 23
5.2. Randomisation 23
6. TRIAL INTERVENTION 24
6.1. Intervention 24
6.2. Control / Comparator 25
6.3. Blinding 25
6.4. Withdrawal from Intervention 26
7. TRIAL PROCEDURES 26

7.1. Data Collection 26
8. END OF TRIAL 27
9. DATA MANAGEMENT 27
9.1. Data Handling 27
9.2. Qualitative Data 29
9.3. Access to Data 29
9.3.1. Access to the final trial dataset 30
9.4. Data Sharing 30
9.5. Study File Management 30
9.6. Archiving 30
10. TRIAL MONITORING AND QUALITY ASSURANCE 31
10.1. Trial Monitoring Plan 31
10.2. Trial Oversight Committees 31
10.2.1. Trial Management Group 31
10.2.2. Trial Steering Committee 31
11. SAFETY REPORTING 31
11.1. Safety Reporting Definitions 31
11.2. Adverse Event Reporting 32
11.3. Adverse Events of Special Interest (AESIs) 33
11.4. Recording and reporting of SAEs and RUSAEs 33
11.5. Responsibilities for Safety Reporting 34
12. STATISTICAL ANALYSIS 35
12.1. Sample Size 35
12.2. Planned Recruitment Rate 35
12.3. Statistical Analysis 35
13. ECONOMIC ANALYSIS 36
14. PROCESS EVALUATION 36
14.1. Identification of intervention mechanisms of action 36
14.2. Patterns of implementation and fidelity 37
14.3. Semi-structured interviews: Acceptability, context and mechanisms of action ... 37
14.4. Recruitment and consent 37
14.5. Interview Schedule 38
14.6. Qualitative Analysis 39
15. ETHICAL AND REGULATORY CONSIDERATIONS 40
15.1. Regulatory Compliance 40
15.2. Research Ethics Committee and Health Research Authority Approval 40
15.3. Amendments 40

15.4. Non-Compliance 40
15.5. Serious Breaches 41
15.6. Ethical Considerations 41
15.6.1. Safeguarding and debriefing procedures 41
15.7. Patient and Public Involvement and Engagement (PPIE) 42
16. CONFIDENTIALITY 43
17. INDEMNITY 44
18. DISSEMINATION 44
18.1. Publication Policy 45
19. REFERENCES 46

ii. LIST OF ABBREVIATIONS

ADHD:	Attention Deficit Hyperactivity Disorder
AE:	Adverse Event
AESI:	Adverse Event of Special Interest
BCT:	Behaviour Change Theory
CEFR:	Common European Framework of Reference
CHU9D	Child Health Utility 9D
CI:	Chief Investigator
CiC:	Community interest company
COMIC:	Child Oriented Mental Health Innovation Collaborative
CONIFAS:	CO-production of a Nature-based Intervention For children with ADHD Study
CRF:	Case Report Form
CTIMP:	Clinical Trial of an Investigational Medicinal Products
CTU:	Clinical Trials Unit
CYP:	Children and Young People
DPIA:	Data Protection Impact Assessment
EDC:	Electronic Data Capture
eTMF:	Electronic Trial Master File
GCP:	Good Clinical Practice

GDPR:	General Data Protection Regulations
GP:	General Practitioner
HHTU:	Hull Heath Trials Unit
HRA:	Health Research Authority
HRQoL	Health Related Quality of Life
HTFT:	Humber Teaching NHS Foundation Trust
ICF:	Informed Consent Form
ICH:	International Conference on Harmonisation
IMD:	Index of Multiple Deprivation
ISF:	Investigator Site File
ISRCTN:	International Standard Randomised Controlled Trial Number
LYPFT:	Leeds and York Partnership NHS Foundation Trust
NCI:	Nature Connection Index
NHS:	National Health Service
NICE:	National Institute for Health and Care Excellence
NIHR:	National Institute for Health Research
NRES:	National Research Ethics Service
PI:	Principal Investigator
PIS:	Participant Information Sheet
PPIE:	Patient and Public Involvement and Engagement
QoL:	Quality of Life
RA:	Research Assistant
R&D:	Research & Development
RCADS:	Revised Children’s Anxiety and Depression Scale
RCC:	REDCap Cloud
RCT:	Randomised controlled trial
REC:	Research Ethics Committee
RfPB:	Research for Patient Benefit
RGF:	Research Governance Framework
RUSAE:	Related Unexpected Serious Adverse Event
SAE:	Serious Adverse Event

SAP:	Statistical Analysis Plan
SEND:	Special Educational Needs and Disabilities
SOPs:	Standard Operating Procedures
TAU:	Treatment as usual
TC:	Trial Coordinator
TMG:	Trial Management Group
TSC:	Trial Steering Committee
UoH:	University of Hull
UK:	United Kingdom
YPAN:	Young Person's Advisory Network

iii. TRIAL SUMMARY

**Study Title**	CO-produced Nature-based Intervention Feasibility for children with ADHD Study (CONIFAS2)
**Short Title**	CONIFAS2
**Study Design**	A multicentre, mixed methods randomised controlled feasibility trial with embedded process evaluation
**Trial Participants**	Children and young people with ADHD
**Planned Sample Size**	84 participants for feasibility randomised controlled trial<br>Up to 20 participants for qualitative interviews
**Follow-up duration (if applicable)**	6 week follow up (42nd day + 14 days)
**Planned Study Period**	20 months
**Research Question / Aim(s)**	To assess if the delivery of the CONIFAS intervention is feasible and acceptable to children with ADHD, their families, and NHS neurodiversity professionals, and to test the feasibility of conducting a randomised controlled trial when compared to treatment as usual.
**Objectives**	1. Feasibility and acceptability of trial processes related to recruitment, randomisation, and collecting health/wellbeing, process and health economic outcomes.<br>2. Feasibility and acceptability of delivering the CONIFAS intervention in neurodiversity services, including CYP experiences of receiving and staff experiences of delivering the intervention.<br>3. How the intervention is embedded into practice to understand how context affects implementation and outcomes.
**Outcome Measures**	PRIMARY OUTCOME MEASURES<br>Feasibility outcomes (below) assessed against pre-defined progression criteria.<br>1. Recruitment<br>2. Retention<br>3. Demographics of participants<br>4. Data completeness<br>5. Acceptability<br>6. Fidelity<br>7. Adherence to intervention<br>CANDIDATE PRIMARY OUTCOME MEASURE FOR FUTURE TRIAL

	Child Health Utility 9D (CHU9D)<br><br>SECONDARY OUTCOME MEASURES<br><br>Collection feasibility of the following measures:<br><br>1. SNAP-IV<br>2. Revised Children's Anxiety and Depression scale (RCADS)<br>3. Nature Connection Index (NCI)<br>4. Bespoke acceptability and accessibility measures<br>5. Bespoke fidelity checklist (clinician-completed)<br>6. Anonymous parent-completed adherence questionnaire<br>7. Intervention daily diary sheet
**Key Terms**	ADHD, Nature, Children, Family support, Quality of life

iv. STUDY FLOW CHART

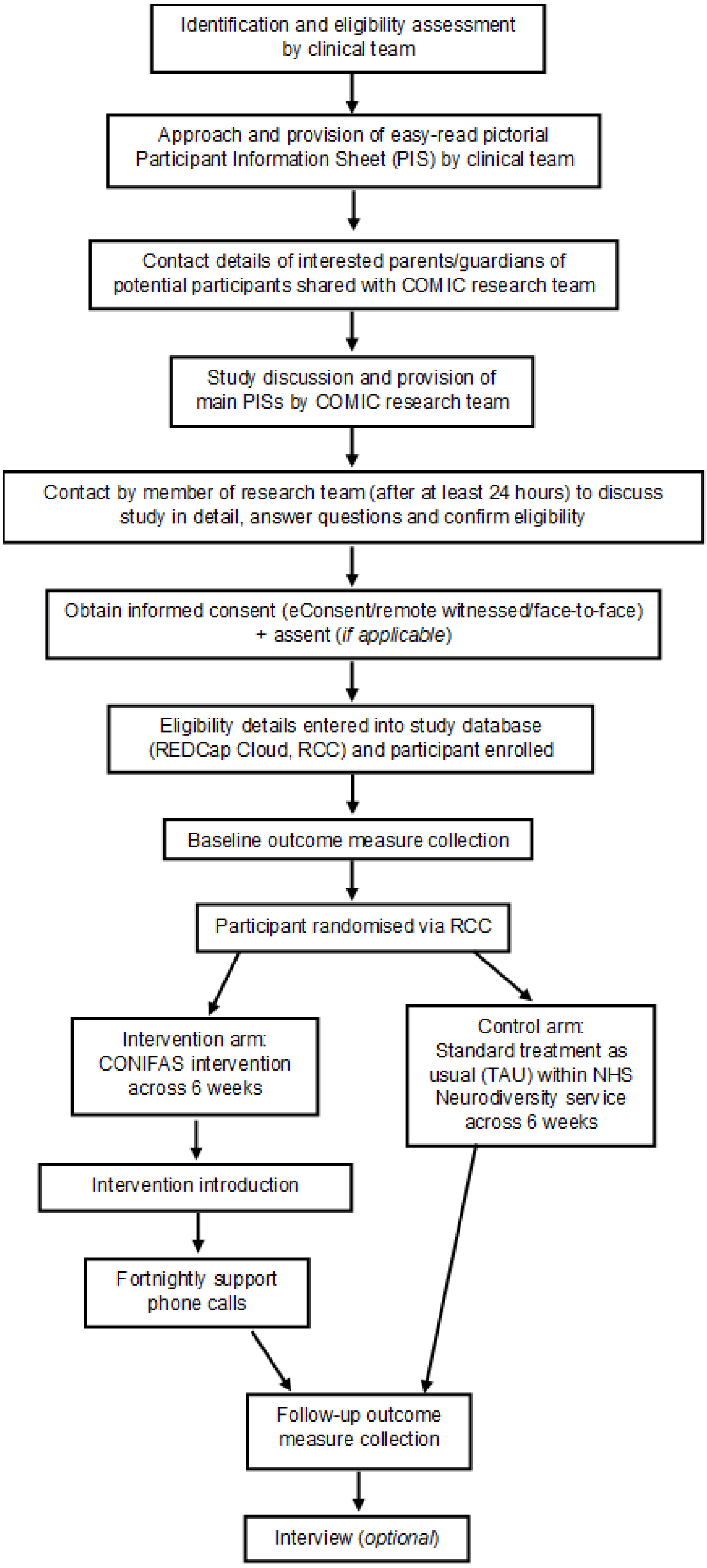

v. SCHEDULE OF EVENTS

ASSESSMENT	SCREENING	BASELINE	+2 WEEKS	+4 WEEKS	+6 WEEKS	+6 WEEKS (+14 DAYS)	END OF STUDY
Identification and eligibility assessment	X						
Study discussion	X						
Eligibility confirmation	X						
Informed Consent		X					
Assent (*if applicable*)		X					
GP letter		X					
Demographics		X					
Medical history		X					
Concomitant medications		X				X	
Usual care details		X				X	
CHU9D		X				X	
SNAP-IV		X				X	
RCADS		X				X	
NCI		X				X	
Randomisation		X					
Intervention introduction session (*intervention arm only*)		X					
10-minute nature activity (*intervention arm only*)		Daily (*if possible*) throughout 6 weeks' trial participation					
Daily diary sheet (*intervention arm only*)		Daily (*if possible*) throughout 6 weeks' trial participation					
Daily sticker chart (*intervention arm only*)		Daily (*if possible*) throughout 6 weeks' trial participation					
Support phone call (*intervention arm only*)			X	X	X		
Intervention acceptability and accessibility questionnaire (*intervention arm only*)						X	
Intervention adherence survey (*intervention arm only*)						X	
Interview informed consent / assent (*optional*)							X
Interview							X

vi. TRIAL ROLES AND RESPONSIBILITIES

Chief Investigator (CI)

The Chief Investigator (CI) will have overall responsibility for the trial design, set-up, conduct, co-ordination and management.

Trial Sponsor

The Sponsor will hold responsibility for overall project management and will be responsible for trial initiation management and financing of the trial as defined by Directive 2001/20/EC. Responsibilities delegated to Hull Health Trials Unit (HHTU) and other parties are detailed in the trial contract and collaboration agreement.

Clinical Trials Unit (CTU)

Hull Health Trials Unit (HHTU) will have responsibility for trial conduct as delegated by the Sponsor in accordance with Good Clinical Practice (GCP) standards. HHTU will conduct trial set-up and monitoring in line with HHTU SOPs, partner SOPs (if applicable) and the research governance framework (RGF). Responsibilities include: trial administration, protocol development, CRF design, trial design, main REC/HRA regulatory submissions, data management, safety reporting, randomisation design and service, database development and provision, database administrative functions, source data verification, monitoring, statistical analyses, HHTU and site training, trial reports and results dissemination.

Trial Management Group (TMG)

The Trial Management Group (TMG) will comprise of the CI, other lead investigators and grant co-applicants (clinical and non-clinical), members of the HHTU team and COMIC research team and a Sponsor representative. The TMG will meet monthly for the first two months of the study to ensure successful study set-up, and then every three months for the remainder of the study, and will be assigned responsibility for trial set-up, trial management, trial promotion, data analysis and publishing trial results.

Trial Steering Committee (TSC)

The Trial Steering Committee (TSC) will comprise of an Independent Chair, not less than two other independent members, and a consumer representative. The TSC will provide overall supervision of the trial looking in particular at trial progress, adherence to protocol, participant safety and consideration of new information. TMG members may attend the TSC meetings to present and report progress. A TSC meeting will be held every 6 months of the study.

1. BACKGROUND & RATIONALE

1.1. Background

Attention Deficit Hyperactivity Disorder (ADHD) diagnoses are associated with socioeconomic deprivation, and diagnosis rates are increasing without an equivalent increase in specialist support services (1). ADHD impacts on daily functioning and outcomes

in multiple domains of health (2). ADHD affects 5% of UK children and families have little access to appropriate, timely interventions post-diagnosis (3). Recent medication shortages have increased pressure on healthcare systems (4). The societal impact of ADHD is significant with the average annual cost of ADHD per child in Europe between $7,369 and $18,616 (2022 prices) (5). A recent review highlighted the need to advance development, evaluation, implementation, and dissemination of evidence-based treatment strategies to support ADHD-related difficulties (6).

Nature-based interventions represent a promising but as yet under-researched approach to supporting children with ADHD in their symptom management and wellbeing (7). Empowering parents and guardians to develop skills and agency to support their children is an important focus of post-diagnostic ADHD provision, particularly for primary age children where indirect means of support are indicated, for example parental support and consultation (8).

This study builds on a successfully completed tier 3 RfPB study called CONIFAS1 (NIHR203043) which co-produced a nature-based intervention for children with ADHD. The intervention, 'CONIFAS' is a home-based, family-led intervention delivered with professional third-party telephone support. It encourages daily engagement with nature for at least 10 minutes, including activity cards and supportive psychoeducational elements. The intervention aims to develop healthy behaviours and support families and children with ADHD to form habits in accessing nature and the outdoors (9). Accessibility was at the heart of the intervention design with the need to ensure families living in more urban areas, and without direct access to outdoor space, could still benefit from the intervention. Research literature indicates small 'micro activities' taking place in urban nature can contribute to positive wellbeing and our intervention has built upon this to include inclusive activities which can be carried out by individuals without access to gardens and larger green spaces (10).

CONIFAS is now ready to be tested in NHS Children's Neurodiversity Services assessing the feasibility and acceptability of a full-scale effectiveness trial.

1.2. Rationale

This research is crucial as it aims to address the significant unmet needs of children with neurodiverse conditions, particularly those with ADHD, who often struggle to access post-diagnostic support in NHS services. In the Hull and East Yorkshire region, over 500 children are awaiting such support, highlighting a critical gap in service provision (Clinical service data). Mental health issues in children and young people, especially those with ADHD, are a growing public health concern, with an 81% increase in referrals for child and adolescent mental health services between April-September 2021 compared to the same period in 2019 (11). Children with ADHD are a particularly vulnerable population with increased rates of mental health diagnosis and need (1).

This study seeks to assess the feasibility of embedding the CONIFAS intervention into existing NHS children's neurodiversity services. CONIFAS, a novel, co-produced intervention, aims to support the well-being and symptom reduction of children with ADHD by providing a green, nature-based approach in addition to existing parent-focused interventions. This approach is timely, as contact with green spaces has been shown to benefit children's physical activity, cognition, behaviour, and mental health (12). Research indicates that even brief exposure to nature, such as 10 minutes outdoors, can improve well-being, with benefits peaking at 200-300 minutes per week (13, 14).

The study also addresses a significant gap in the research related to inequalities in access, engagement, retention, and outcomes of ADHD interventions, as highlighted by the NIHR (2019). By evaluating the feasibility of the CONIFAS intervention in a real-world context, this research will provide important data to inform future larger-scale evaluations. Furthermore, by collecting health economic data, the study aims to assess the potential social and economic impact of the intervention on ADHD treatment.

The research builds on existing evidence supporting the efficacy of psychosocial interventions for children with ADHD, such as parent training and behavioural interventions (6, 8). During consultation with stakeholders from Navigo Community Interest Company (CiC) and Humber Teaching NHS Foundation Trust (HTFT), there was positive support for the integration of CONIFAS alongside NICE-recommended interventions, emphasising the importance of empowering parents to support their children in neuro-affirming ways.

Following the Medical Research Council's (MRC) guidance on complex intervention development (15), the study will collaboratively develop a programme theory using behaviour change theory and relevant frameworks (16, 17). This will inform the intervention's refinement and scalability, supporting its wider implementation in future effectiveness trials.

2. AIMS AND OBJECTIVES

The overarching aims are to assess if the delivery of the CONIFAS intervention is feasible and acceptable to children with ADHD, their families, and NHS neurodiversity professionals, and to test the feasibility of conducting a randomised controlled trial when compared to treatment as usual.

The objectives are to address uncertainties regarding:

- Feasibility and acceptability of trial processes related to recruitment, randomisation, and collecting health/wellbeing, process, and health economic outcomes.
- Feasibility and acceptability of delivering the CONIFAS intervention in neurodiversity services, including children and young people's (CYP) experiences of receiving and staff experiences of delivering the intervention.
- How the intervention is embedded into practice to understand how context affects implementation and outcomes.

2.1. Trial Outcomes

As a feasibility trial, the main outcomes are related to the feasibility / process evaluation. The intended trial outcomes can be grouped together:

1. **Feasibility outcomes and process evaluation**

Data will be collected to inform the optimum design and processes of a future RCT.

- **Recruitment:** Is it possible to recruit to the study? Number of eligible young people, number approached, number participating, number of exclusions due to non-English speaking families – what are the common languages and how can participation be enabled in a larger study?
- **Retention:** Do participants withdraw from the study? If so, why, and how can withdrawal rates be reduced?

- **Demographics of participants:** Is it possible to recruit a diverse sample, and can balance be achieved across groups – age, gender, ethnicity, socio-economic status (parent demographics and postcode), Special Educational Needs and Disability (SEND) status, comorbid conditions, medication use, service wait times including referral point and previous Children's Neurodiversity Service Involvement?
- **Data completeness:** Do parents and children complete the outcome measures? Missing data, completion rates, differential completion between arms.
- **Acceptability:** Is the intervention, and an RCT design acceptable from the perspective of families, children and staff, assessed qualitatively through semi-structured interviews?
- **Fidelity:** Can the intervention be delivered with fidelity by NHS staff?
- **Adherence to intervention:** Will children and parents/guardians engage with the intervention?

2. Programme Theory

The first iteration of the programme theory will be refined with data collected in semi-structured interviews.

2.1.1. Outcome Measures

Primary outcomes

The primary outcomes of this study are the feasibility outcomes. These outcomes will be assessed against pre-defined progression criteria, which have been mapped to key feasibility objectives. Current status will be reported to the trial oversight committees using a traffic light system as defined in the table below. Key stakeholders will be involved in the final assessment and discussions about whether to proceed to development of an application for a definitive RCT, using the decision and reporting (18).

Criteria	GREEN (progress to large-scale RCT)	AMBER (consider changes to intervention or trial design)	RED (do not progress – intervention delivery or RCT not feasible)
**Site set-up:** Site approvals obtained, staff trained to deliver intervention, open to recruitment (site greenlight)	2 or more sites fully achieve criteria	1 site achieves criteria, important insights from other sites to inform how to overcome challenges for a future study	0 sites meet criteria, not possible to overcome challenges
**Recruitment rate:** Aim to recruit 84 participants across sites over 9 months	Average 5 or more participants recruited per site per month	Average 3-4 participants recruited per site per month	Average <3 participants recruited per site per month
**Fidelity of intervention delivery:** Intro session and weekly calls occurred and content delivered	All core components of the intervention delivered	>75% of core components delivered	<= 75% of core components delivered
**Engagement / adherence with the intervention:** Attendance at intro session, fortnightly calls x3, completion of daily intervention tasks	Attendance at intro session and all calls, completion of activities >80% of intervention tasks	Attendance at intro and 2/3 calls, completion of 50-80% of intervention tasks	Attendance at intro session and 1/3 calls, completion of <50% of intervention tasks
**Acceptability of intervention / RCT:** Qualitative process evaluation	No major concerns identified	Some acceptability issues identified that can be addressed with adaptations / refinement to the intervention or study design	Major acceptability issues that would require substantial re-design of intervention or study
**Data collection:** Data completion rates, CHU9D (candidate primary outcome) at baseline and 6 weeks	85-100% completion rate (allowed for 15% attrition in sample size)	60-84% completion rate	<60% completion

Candidate primary outcome for future trial

The candidate primary outcome for a full trial is the CHU9D (19). This is a 9-item questionnaire which enables measurement of improvements to wellbeing (identified as a central concern in Patient and Public Involvement and Engagement (PPIE) work). In a study of 455 families containing 476 children diagnosed with ADHD, children diagnosed with ADHD were found to have a CHU9D score around 6% lower than comparable children without ADHD (20). As a generic preference-based measure of health-related quality of life (HRQoL) it is also suitable for assessing value for money and informing decisions about which health interventions should be publicly funded. This will be assessed by proxy completion for those under 7 years, and either proxy completion or self-report for children 7+ years depending on preference.

Secondary outcomes

The secondary outcomes are all related to the measures proposed to be collected in a full trial. These are not measured in this study to examine effectiveness but will be used to judge collection feasibility for a full trial. The child and parent/guardian burden to complete all measures and accessibility have been considered with PPIE. Measures to be collected include:

- SNAP-IV 26-item – a Parent Rating Scale for ADHD symptoms (parent/guardian-completed).
- Revised Children's Anxiety and Depression Scale (RCADS) - a 47-item questionnaire with subscales (parent/guardian-completed).
- Nature Connection Index (NCI) - a 6-item measure which gathers evidence on the relative importance of contact with nature and nature connection to the individual (child completion).
- Bespoke acceptability and accessibility (of the intervention in terms of language used throughout and difficulty level of suggested activities) measures used in CONIFAS1 (22) (parent/guardian, child, and clinician completion).
- A bespoke fidelity checklist (clinician completed) and an anonymised parent-completed questionnaire via RedCap Cloud to assess adherence to the intervention.
- Daily diary to measure frequency and duration spent engaging in CONIFAS intervention and activity or activities completed (parent/guardian completed).
- Daily sticker chart to record activity (Child completed)

Uptake and implementation

The frequency and duration of engagement with the CONIFAS intervention will be assessed. As this is a complex intervention, adopting conventional measures of 'dose' are unlikely to be consistent across the population of interest. As such, data will be collected on the number of days and time spent engaging in outdoor and nature activity using the CONIFAS intervention, number of sessions attended, and number of dropouts from the intervention. These data will enable identification of the frequency and duration of uptake of the intervention, providing critical information about the range of engagement across the population. A simple diary sheet for families to record the time spent engaging in CONIFAS activities per day, how often they took part in activities, and what activity they completed will be included. The diary format will be kept simple as user feedback from the first CONIFAS1 study indicated that the longer reflective diary used was not completed frequently by parents.

3. TRIAL DESIGN

The study will be conducted in compliance with the protocol, Good Clinical Practice (GCP) and UK research regulations.

3.1. Study Design

This is a multi-centre, randomised controlled feasibility trial comparing the CONIFAS intervention to 'Treatment As Usual' (TAU) when provided to families on the post-diagnosis ADHD support pathway. TAU is defined as the current provision of the site from which participants are recruited. This will be recorded throughout the study. The study also includes a feasibility health economics evaluation and a process evaluation.

The recruitment target for the study is 84 participants who will be randomly allocated to the two study arms. Participants will be expected to take part in the study for a total of 6 weeks.

3.2. Study Setting

The study will be conducted across four children's neurodiversity ADHD services who will be the four sites for the study. These are:

Humber Teaching NHS Foundation Trust (HTFT)

Navigo Community interest Company (CiC) who hold the NHS contract for ADHD service delivery in North-East Lincolnshire.

Leeds Community Healthcare NHS Trust (LCH)

Cornwall Partnership NHS Foundation Trust (CFT)

Once recruited, intervention delivery for those randomised to receive the intervention will take place in their homes and local areas.

4. RECRUITMENT

4.1. Eligibility

Eligibility to participate in the trial will be initially assessed by the Site Principal Investigator (PI) (with support from the NHS Trust's research team if required), and will be determined in accordance with the following eligibility criteria. The CONIFAS2 research team will then confirm eligibility at first contact with the participant. Eligibility waivers are not permitted by the National Research Ethics Service (NRES).

4.1.1. Inclusion Criteria - Child

Participating children must:

- Be aged 6-11 years
- Be on an open clinical caseload for intervention or waiting list post-diagnosis
- Have sufficient understanding of English to participate in the study
- Have parent(s) / guardian(s) with sufficient understanding of English to participate in the study

4.1.2. Exclusion Criteria – Child

Participating children should not:

- Pose a risk of harm to self or others when under parental supervision

- Have profound additional difficulties as determined by parent(s) / guardian(s)

Children with comorbid diagnoses (e.g. autism, physical difficulties) will be supported to participate with recognition that additional considerations in accessing outside spaces may arise. Any relevant risks will be assessed during the consent procedure.

For each child participant, a parent/guardian will also be recruited for completion of outcome measures.

4.1.3. Inclusion Criteria – Parent/Guardian

- Is a parent/guardian for a child study participant
- Has sufficient understanding of English to participate in the study
- Is willing and able to complete the study outcome measures

4.1.4. Exclusion Criteria – Parent/Guardian

- Is unwilling or unable to consent or complete the study outcome measures

Sufficient understanding of English has been defined using standard definitions provided by the Common European Framework of Reference (CEFR) to include those with intermediate English skills. Those who have intermediate skills can understand information about familiar topics. They can communicate in most situations whilst travelling in an English-speaking area. They can write simple connected texts on familiar topics. This would enable them to read and understand the study materials with support from the professional and complete outcome measures.

An easy-to-read information sheet has been created and support will be provided to complete outcome measures which may contain more complex language to make the study as inclusive as possible. Additional languages required will be recorded and considered for inclusion in a full trial.

4.2. Screening

Initial identification and screening will be undertaken by the clinical teams within NHS Neurodiversity Services. Teams will review eligible service users at the end of the assessment pathway, treatment pathway or waiting list and will log all screened service users on a screening log. The screening log will include anonymised information on gender, age, ethnicity, postcode (for calculation of Indices of Multiple Deprivation (IMD)), comorbidities and the reason for ineligibility (if applicable). The screening log and recruitment information will be stored in a restricted access folder on Box collaborative access cloud file storage system (see Section 9 for further details).

Parents/guardians of eligible potential participants will be approached by the clinical teams and provided with verbal and written information about the study during a routine clinic or phone call. Those that express an interest in the study will be asked if they consent for their name and contact details to be shared with the CONIFAS2 research team. If parents/guardians of potential participants prefer not to share their contact details, the participant information sheet (PIS) will include contact details for the research team so that they can initiate contact if they wish to take part in the study.

Any reasons for non-participation will be documented on the screening log.

4.2.1. Enhancing Equality and Diversity

As part of the intervention development work during the study, it is anticipated that versions of the intervention in several of the most prevalent languages used in the local region (i.e. Polish and Urdu) will be devised to support inclusion of non-English speaking families.

In addition, the potential use of AI-assisted translation tools for audio versions of the nature activity box elements and support videos (e.g. introduction information videos) will be explored, as well as a version with greater reliance on pictorial representations and less text, as will the costs for validation of some of the outcome measures (e.g. SNAP-IV and NCI) in other languages. Patient and public involvement and engagement (PPIE) groups will also be utilised to work closely with a range of individuals to plan how the intervention could be utilised for a diverse range of children and families.

4.3. Informed Consent

The CI retains overall responsibility for informed consent of participants and must ensure that anyone delegated responsibility to conduct informed consent is duly authorised, trained and competent to undertake consent according to the ethically approved protocol and Good Clinical Practice (GCP). Informed consent must be obtained prior to the participant undergoing any non-routine study specific procedure.

There will be three PISs for the study to be provided to families: the main study PIS for parents/guardians, an easy-to-read pictorial based PIS, and an easy read PIS for children. The easy-read pictorial-based PIS will be provided to families by clinicians at participating sites in order to gauge initial interest in the study. A member of the CONIFAS2 research team will contact the parent/guardian of eligible and interested individuals passed on by the four clinical sites. They will explain the study and provide the main study participant information sheets (PISs; parent/guardian and child versions). Clinical teams may follow up with families to confirm interest in the event of repeated non-response to contact attempts by the CONIFAS2 research team.

Families interested in participating in the study will be asked to read the PIS and to discuss the child PIS with their child. Parents/guardians and potential participants will be given ample time to consider their participation in study (usually a minimum of 24 hours) before being contacted again by the researchers who will confirm eligibility with the parent/guardian, confirm understanding of the study requirements, and answer any questions the family may have.

If they would like to proceed, parents/guardians will be asked to complete consent forms which provide consent for theirs and their child's participation in the study. The parent/guardian must have verified legal responsibility to provide consent for their child. Children will be invited to complete a simple assent form if they express interest in participating, recognising that parental consent provides the legal basis for inclusion. Once a parent/guardian has provided consent on behalf of a child, a letter will be sent to the child's GP to inform them of their participation in the trial.

In addition, a video containing study information and details on the consent procedure will be available for any parents/guardians with poor literacy skills who may be unable to read or sign the consent form or may have problems with comprehension. This method of providing information may also be preferable for any parents/guardians who are neurodivergent themselves. Those viewing the video will also have the option to access the full PIS and will be asked to confirm they have viewed and understood the video and are happy to proceed

as part of the consent process. The key study details will be reiterated and understanding of these confirmed as part of the consent discussion with the researcher.

Consent for this study will be received by appropriately trained members of the CONIFAS2 research team as delegated by the CI on the Delegation Log. Consent will primarily be completed via e-consent or remotely witnessed verbal consent however a face-to-face visit will be arranged if requested by a parent/guardian. Assent forms can also be completed using the same methods. Parent/guardian email addresses will be used for the child to provide assent electronically.

Remote consent – eConsent

eConsent will be conducted via Box Sign, integrated with the HHTU Box account. The researcher initiates the process using a web form, entering the parent/guardian's and their own email addresses. Guided by the researcher during a phone/video call, the parent/guardian receives an email with a link, initials consent statements, and signs the form. The researcher then countersigns. Once completed, signed electronic copies are automatically sent to both parties and the study site by email.

If participants are not able or willing to complete the consent via Box Sign, consent will be obtained over a telephone or video call and documented by the researcher. Each informed consent form (ICF) statement will be read aloud to the participant and their parent/guardian and verbal agreement confirmed prior to the researcher initialling each statement. Consent will be witnessed by a second member of the research team. Both the researcher and the witness will sign the ICF via Box Sign. Face to face consenting appointments will also be provided at the request of the participant. Where possible Box Sign will still be used at these appointments however if this is not possible due to technology not being available a paper form will be completed. Consent for those who have viewed the video PIS will be remote witnessed consent only.

All consent methods

All consent forms will be stored in accordance with local requirements. A copy of each completed consent form (i.e. for the parent/guardian and for their child) will be provided to the participant's parent/guardian. The original form should be filed in the Investigator Site File (ISF), and a scanned copy added to the participant's clinical record and uploaded to the trial database held on REDCap Cloud (RCC) for central monitoring purposes.

Eligibility and consent will be documented on a consent log/participant register. This will contain personally identifiable information and will be stored separate from collected research data. The consent log/participant register will contain the unique identifier which will be automatically assigned at the point of enrolment into the main study database and will be used throughout the study to ensure that data is pseudonymised. Consent will be obtained to collect and store both personally identifiable information and data.

4.4. Participants who Choose Not to Participate

The right of a participant to decline participation without giving a reason must be respected.

4.5. Withdrawal of Participants

Each participant must remain free to withdraw at any time from the study without giving reasons and without prejudicing their future care. The investigator(s) or sponsor may withdraw participants from the study only if indicated by a safety/clinical issue.

Where a participant is required to re-consent or new information is required to be provided to a participant it is the responsibility of the PI to ensure this is done in a timely manner and according to any timelines requested by HHTU. The CONIFAS2 research team and site NHS Research and Development teams can also support the PI in this process. The PI takes responsibility for ensuring that all vulnerable participants are protected and participate voluntarily in an environment free from coercion or undue influence.

Any withdrawals will be documented on the consent log. Where possible, reasons for withdrawal will be obtained from participants. It will be important to collect this information to identify whether there are any procedures that may need to be amended for a full trial.

5. ENROLMENT AND RANDOMISATION

5.1. Enrolment

Once informed consent has been obtained, eligibility details, including confirmation of e-consent, verbal or in some circumstances written consent, will be entered into the Eligibility electronic Case Report Form (eCRF) on the RCC study database by an authorised member of the CONIFAS2 study team in order to enrol the participant onto the trial. This will automatically generate a pseudonymised Participant ID Number, which will be used on all study documentation to identify the participant for the remainder of the study. Participant ID Numbers will be created according to the format: CF2-01-001 (CONIFAS2, Site 01, Participant 1).

5.2. Randomisation

A web-based randomisation system will be provided by Hull Health Trials Unit (HHTU), within a commercial web-based system, REDCap Cloud (RCC). Participants will be randomised in a 1:1 ratio using random permuted blocks to study arm (i) CONIFAS intervention, or study arm (ii) TAU, stratified by site. The randomisation sequence will be prepared by an independent HHTU statistician. All trial staff and sites will remain blinded to the sequence. Prior to recruitment starting, a test system will be made available to the research team for training purposes. Any user comments or suggestions on the usability of the system will be fed back to HHTU before the system is made live.

Randomisation will occur after eligibility has been established, informed consent has been obtained, and baseline measures have been collected from participants and their families. The allocation schedule will be concealed to research staff, with the study arm only confirmed once participant enrolment is complete.

Authorised members of the CONIFAS2 research team will access the online randomisation system within RCC and enter the required participant details to request randomisation. These researchers will receive immediate notification that the randomisation has been performed but will remain blinded to the allocation results. HHTU will also receive an alert with confirmation of the participant randomisation and assigned Participant ID Number. Once a participant has been randomised, the online randomisation system will automatically notify the Trial Coordinator (TC) at the Child Oriented Mental Health Innovation Collaborative (COMIC) in Leeds and York Partnership NHS Foundation Trust (LYPFT) via email of the unblinded allocation to treatment. The TC will then notify the clinical sites of the allocation and arrange distribution of the intervention boxes as appropriate.

6. TRIAL INTERVENTION

6.1. Intervention

Description: CONIFAS nature-based intervention

Rationale: The CONIFAS intervention was co-produced as an intervention to support children with ADHD and their families to engage with nature to improve quality of life and reduce ADHD symptoms. It is a home-based, family-led intervention with support from a professional (21) and optional peer support.

Materials: The activity box element of the intervention is presented in a portable box and includes: 1) 47 laminated '10-minute challenge' activity cards, colour coded by seven categories (rules and boundaries, active, body and senses, creative, discovering, relaxing, and exploring); 2) 36 'Can you find something' cards; 3) a psychoeducation booklet for parents / guardians; 4) a psychoeducation and activity booklet for children. The intervention box also includes a mini torch, modelling clay, a wood 'cookie' to draw or paint on, and a daily challenge calendar with nature stickers.

Procedures: The intervention box will be provided to families randomised to the intervention either face-to-face or via the post. Once the box has been received, a neurodiversity ADHD service clinician will provide an initial introduction to the intervention (psychoeducation and practical advice), most likely via telephone or video call, and then a fortnightly support call (three in total over the six-week intervention period) for encouragement and problem solving. Two videos are available to support families' introduction to the intervention, as well as QR codes for audio recordings of written text. Supporting documentation for the fortnightly calls was developed with a PPIE group and stakeholders. Families of participants randomised to the intervention group will also have the option of joining a community of experience forum for additional peer support.

Community of experience forum: In response to PPIE feedback, a moderated (by Kids organisation) Facebook-hosted community of experience forum for all parents or guardians of those taking part will be offered to all those randomised to the intervention arm. Throughout the co-production and user testing parents and guardians noted that a peer support element and opportunity to connect with other families would be invaluable in supporting them in using the intervention. This aspect of the intervention will be analysed as part of the behaviour change work (see Section 14.1).

The Facebook forum will be optional, and parents/guardians will be able to indicate on the consent form whether they give consent for a link to the forum being shared with them (if they are assigned to the intervention arm). If they indicate yes and are then randomised to the intervention arm, the TC will send a link to the forum. Parents/guardians will be made aware on the consent form that by joining the forum personal details will be made visible to the moderator and the other members. All content shared in the forum will first be checked and approved by the moderator (Moderator from the Kids organisation). Participants will need to enter their participant ID in order to gain access to the forum as well as answer a question to verify their involvement in the study for example site and name of clinician supporting them. Participants will also be asked to agree to a clause respecting the confidentiality of other forum users.

Setting: The intervention will be carried out in local green spaces around the children and families' homes, for example in gardens and parks, though they are also welcome to access nature spaces further afield. Families are encouraged to access nature for ten minutes every day. This can be done anywhere that is available to them, there is no need to travel far to

access countryside, etc. Some of the nature activities can be done inside the home whilst using nature (e.g. nature listening through an open window).

Delivery will take place over six weeks with families encouraged to undertake at least ten minutes of daily nature engagement in whatever way they would like to each day. The ten-minute dose of nature was designed to be a practical and achievable time for parents to spend time each day outdoors based on discussions with parents/guardians during the CONIFAS1 workshops.

Tailoring: The CONIFAS intervention has been developed with accessibility in mind. During co-production, it was considered how families who live in apartments with reduced access to green space or those living in more urban areas can benefit from the intervention. There are a number of activities which take place in the home using natural materials or which do not need significant resources (e.g. cloud watching, listening to birds). Psychoeducational components of the intervention provide an understanding of the evidence base for parents/guardians including the fact that small 'micro-activities' in nature can be beneficial for wellbeing.

6.2. Control / Comparator

All participants will continue to receive Treatment as Usual (TAU) according to their current site provision. The components of TAU will be recorded for each individual participant and whether this changes over the course of the study. Current commissioning for both services is to be placed on a waiting list with crisis management as required. No medication is prescribed until children have received a medical assessment. Should the services change their treatment pathways, discussion will be had with sites about how CONIFAS can continue to fit as part of a wider intervention pathway, and any changes in participant care will be recorded. At the end of the study the control group will receive a nature-themed goodie bag which will include elements of the intervention, i.e. activity cards and starter activities.

6.3. Blinding

Members of the CONIFAS2 research team who perform the baseline and follow-up outcome measure data collection will remain blinded to allocation where possible. Baseline outcome measures will be collected prior to randomisation in order to eliminate the risk of initial unblinding.

In order to reduce the risk of unblinding during the follow-up calls, specific wording will be agreed in advance to be used by the researcher at the start of each call to alert the participant's parent/guardian of the need to keep the allocation concealed during the call.

However, as intervention arm participants will be asked to complete the intervention acceptability questionnaires during the follow-up call, this will require unblinding of the researchers so they know whether these questionnaires need to be completed with the participant. To minimise the chances of earlier unblinding, the acceptability questionnaire eCRFs will be hidden until the researcher selects the 'Unblinding' option at the end of completing the other follow up measures which are common to both groups. The researchers will be instructed to unblind only once the rest of the outcome measures have been completed.

The researchers will also remain blinded to the specific details of any serious adverse events (SAEs) reported during the study as these will be assessed for relatedness to intervention. The occurrence of an SAE will be visible to the researchers on the study database, but

access to the eCRF containing details of the SAE will be restricted and cannot be viewed by the researchers.

The extent to which blinding can be maintained within the study will contribute to the assessment of study feasibility.

6.4. Withdrawal from Intervention

Participants may choose to stop taking part in the intervention at any time but will still have the option to complete outcome measures at the 6-week follow-up point if they wish to. This will be confirmed with them when they notify the team of stopping the intervention.

Participants that choose to withdraw consent from the intervention and/or further data collection will be reminded that data collected up to that point will remain on file and will be included in the final study analysis but that it will be anonymous and not identify them in any way. If participants continue to express concern about having their data used it may be excluded from data analysis wherever possible unless it is deemed to have a significant negative impact on the scientific value of the study findings. All participant study documentation will be kept for monitoring and inspection purposes until the study documentation archiving period has ended in line with regulatory body guidelines.

7. TRIAL PROCEDURES

7.1. Data Collection

Data collection for baseline outcome measures will be carried out during the same telephone/video call as the consent call wherever possible. Should participants require a separate appointment for outcome measure completion, this will be arranged. If a participant requests, face-to-face visits can also be accommodated. This will be considered on an individual basis.

Baseline data will be collected by the blinded CONIFAS2 researchers and will include the following demographic information: age, gender, ethnicity, socio-economic status (parent/guardian demographics and postcode), Special Educational Needs and Disability (SEND) status, comorbid conditions, medication use, service wait times including referral point, and previous Children's Neurodiversity Service involvement. Parent/carer relationship to the child will also be confirmed.

The baseline measure for the candidate primary outcome for the future trial is the Child Health Utility, CHU9D. This will be self-reported for participants 7+ years (or proxy completed if preferred) and proxy completed by the parent/guardian for those under 7 years.

Baseline measures of all secondary outcomes (SNAP-IV, RCADS, NCI) will also be collected. The SNAP-IV and RCADS questionnaires will be completed by the parent/guardian, while the NCI will be completed by the child. If the child does not wish to be present on the call, but is happy to provide answers to the NCI, the parent/guardian will be asked to send their answers via email within 24 hours of the call.

All baseline outcome measures will be created in questionnaire format within the RCC study database. Blinded CONIFAS2 researchers will enter the data in real-time whilst on the call with the participant's parent/guardian. Support can be provided to complete the outcome measures either online, face-to-face or via telephone as required.

Baseline outcome measures are expected to take approximately 30-60 minutes to complete (parent/guardian and child measures).

A repeat of all candidate outcome measures will be undertaken at 6-weeks post-baseline or up to two weeks after this date. Additional bespoke questionnaires assessing acceptability and accessibility of the intervention in terms of language used throughout and difficulty level of suggested activities will be completed at follow-up by parents/guardians and children. Similarly to the baseline data collection, parents/guardians can provide their child's answers to the NCI and to the child's Acceptability Questionnaire via email within 24 hours of the follow-up call.

Participants will also be asked to complete a daily diary to measure the frequency and duration of engagement with the CONIFAS intervention and types of activity or activities completed, as well as the CONIFAS activity calendar, a simple sticker sheet to record if an activity has been completed each day. Participants will be asked to return this to the research team at the end of their participation in the study. This will be entered into the database by the research team.

A subset of participants from both the control and intervention arms will be approached to participate in the qualitative arm of the study. Further details on this can be found in Section 14.3.

A bespoke fidelity checklist (clinician completed) will be utilised to record the content of introductory session and fortnightly calls, and an anonymised parent-completed questionnaire via REDCap Cloud will be used to assess adherence to the intervention. A paper version will be provided with a stamped addressed envelope for postal return for any participants unable to complete the online questionnaire.

8. END OF TRIAL

The end of the trial is defined as the completion of the last participant interview.

An End of Trial Declaration Form will be submitted to the main REC, Sponsor and site R&D within 90 days from the End of Trial date or within 15 days if the trial is discontinued prematurely. The CI will ensure that any appropriate follow up is arranged for all participants. A final summary report will then be submitted to the REC, Sponsor and site R&D within 12 months of the end of trial date.

Site R&D will be notified immediately of any reason to halt the trial. The CI and Sponsor will decide if the trial should be halted temporarily. The REC and site R&D will be notified within 15 days of a decision to temporarily halt the trial by submitting a substantial amendment notification.

9. DATA MANAGEMENT

9.1. Data Handling

HHTU will develop the study database and data processes in accordance with HHTU SOPs. HHTU data systems are within scope of the HHTU NHS Data Security and Protection Toolkit (Organisation Code – EE133824-HHTU). All of the information collected about the participants in the course of the trial is confidential and will be held in accordance with GDPR 2018.

A trial-specific Data Management Plan agreed by the CI, Sponsor, HHTU and study statistician will be drafted to provide detailed instructions and guidance relevant to database set up, data entry, validation, review, query generation and resolution, quality control

processes involving data access and transfer of data to the Sponsor at the end of the study, and archiving.

Data will be collected and stored in an online electronic data capture (EDC) system managed by HHTU (REDCap Cloud (RCC)). RCC is a cloud-based EDC system provided by nPhase. Data are stored on dedicated RCC hardware in EU datacentres (including real-time backup) managed by Amazon Web Services to industry standards outlined in ISO 27001, PCI DSS, SOC 1-3, FISMA, CIS, CSA, NIS and UK Cloud Security Principles. Data are encrypted at rest and in transit. RCC themselves deliver compliance to HIPAA, CFR Part 11 and EMEA Annex 11.

Online electronic case report forms (eCRFs) within the study database will be used for data capture for individual participant data required by the trial protocol, identified by unique Participant ID Number. The design of the eCRFs will:

- Enable adequate collection of data
- Provide an audit trail to demonstrate the validity of the trial (both during and after the trial)
- Ensure that only the data required by the protocol are captured

The participating sites and CONIFAS2 research team will retain a copy of any source data entered on the eCRF to ensure that the PI can provide access to the source documents to a monitor, auditor or regulatory agency to check for any transcription errors.

Granular role-based permissions will ensure members of the study team can enter and view the information required by the protocol. Access to the EDC system is managed by the HHTU Data Management team. Users will be required to complete training and sign a terms of use document. All activity within RCC is subject to a full user audit, including timestamp.

Participant consent forms (fully identifiable) will be stored securely in the Box collaborative cloud file storage system (if eConsent) until the study archiving retention period is reached, at which point they will be deleted. Any paper consent forms will be stored securely by the CONIFAS2 researchers in a double-locked location and archived with other source documents in accordance with local procedures.

Qualitative interview recordings and transcriptions will also be stored securely using the HHTU Box collaborative cloud file storage system and/or securely in COMIC MS Teams on OneDrive if completed this way. The HHTU Box instance is administered solely by HHTU staff with access limited to HHTU staff and authorised members of the research team. Box uses EU data centres and complies with (and is independently audited against) the following standards:

- SOC 1, 2 and 3
- ISO27001/270018
- Department of Defence Cloud Computing Security Requirements Guide
- FedRAMP Level 4
- FISMA
- FINRA/SEC 17a-4
- HIPAA/HITECH

Further details on Box data security and General Data Protection Regulations (GDPR) compliance is available here: https://www.box.com/n-gb/security. The data protection risks of HHTU's use of Box have been formally evaluated via a data protection impact assessment (DPIA).

The CI and study staff involved with this project will comply with the requirements of the GDPR and the Data Protection Act 2018. The HRA recommended wording to fulfil transparency requirements under the GDPR for health and care research has been included in the PIS. In line with the Data Protection Act (2018), access to fully identifiable information will be minimised. For the purposes of ongoing data management, once created as a participant on the RCC database, individual participants will only be identified by their unique Participant ID Number on all subsequent forms and documents. Data is therefore pseudonymised and as such will still be treated as personal data.

The analysis datasets will contain only pseudonymised data (Participant ID Number) but the team will have no access to the key. As such, the data will be classed as anonymous in context.

Data will be monitored for quality and completeness by HHTU. Missing data will be chased until it is received or confirmed as unavailable. Missing data items will not be chased from participants.

Following data completion and resolution of queries, the database will be locked. Data will be exported from the trial database in the required formats for analysis, long-term storage and sharing. The trial dataset will be exported from RCC for analysis and stored in HHTU Box Instance with restricted access. HHTU SOPs will be followed for the process of database lock and export including pseudo-anonymisation. HHTU will ensure no direct participant identifiers are present in any exported datasets.

The locked study dataset will be transferred to the statistician team for analysis at the end of the trial. Each site will receive a copy of their locked dataset before the final analysis takes place. Analysis datasets will be transferred using HHTU Box, with a specific secured folder created. Access will be granted to the statistician team. Further details regarding data storage and transfer are provided in the HHTU Data Protection and Confidentiality SOP.

All hard copy trial documents will be retained at study sites in a secure (locked when not in use) location during and when the trial is finished in accordance with data protection requirements for the retention of research data and local site data management policies.

9.2. Qualitative Data

Qualitative interviews will be recorded on encrypted Dictaphones or Microsoft Teams, and audio/video files uploaded to a secure HHTU Box folder. Audio/video files will be transcribed verbatim by a University of Hull (UoH)-approved transcription company. Transcripts will be labelled with unique identifiers and stored in Box. Audio/video-recorded data will be destroyed once transcription is completed and the allocation of unique identifiers have been assigned.

9.3. Access to Data

Direct access will be granted to authorised representatives from the Sponsor, host institution and the regulatory authorities to permit trial-related monitoring, audits and inspections.

HHTU Data Management Team will control access permissions to the RCC database and the eTMF held on Box throughout the trial and afterwards when the documents have been archived. Access permission will be granted based on delegated trial role and level of access required, e.g. editor or read only. Access will be revoked when staff leave the trial or at the end of the trial after data lock.

9.3.1. Access to the final trial dataset

The database will be ‘locked’ to obtain the final dataset after trial completion (completion of final participant interview); completion of coding and data entry; all data queries resolved and the database updated; and serious adverse event (SAE) queries resolved and the database updated; and the trial team notified of date of lock.

A copy of the final trial dataset and notification of completion of the trial participant follow-up and interviews will be sent to the Sponsor before the randomisation list will be released by HHTU prior to the statistical analysis. A copy of each individual site’s data will be sent to that site. A copy of the final trial dataset will be archived by HHTU. Participant consent forms held on RCC will not be exported by HHTU as part of the final trial dataset.

9.4. Data Sharing

Anonymised participant data will be made available on request following consideration by the study team on a case-by-case basis. Requests should be made to the CI and will be considered by the TMG. Clinical information will not be released without the written permission of the participant, except as necessary for monitoring and auditing by the Sponsor or its designee. The CI and study staff involved with this study will not disclose or use for any purpose other than performance of the study, any data, record, or other unpublished, confidential information disclosed to those individuals for the purpose of the study. Prior written agreement from the Sponsor or its designee will be obtained for the disclosure of any said confidential information to other parties.

9.5. Study File Management

The trial will use an electronic Trial Master File (eTMF) which will be held on Box and managed by the HHTU Data Management Team. Access will be restricted and will be granted based on role and level of access required.

Site research teams will be expected to maintain a file of essential trial documentation (an Investigator Site File, ISF) provided by HHTU and keep copies of all completed documents for the trial. All trial files will be stored in accordance with GCP guidelines and GDPR 2018 regulations.

9.6. Archiving

Archiving will be authorised by the Sponsor following submission of the End of Trial Report. All essential trial documents, including source documents where possible, will be archived in accordance with the HHTU Data Management Plan and Archiving SOP for a minimum period of ten years after trial completion. Destruction of essential documents will require authorisation from the Sponsor.

The archiving of trial documents and data at site (including consent forms) will be done according to participating site Trust policies and SOPs. HHTU will archive the trial eTMF and datasets in line with HHTU SOPs. The trial eTMF and datasets will be moved to a restricted access folder within Box, managed by HHTU on behalf of the Sponsor. Document retrieval from the archive will be documented and will be fully auditable.

HHTU have backup procedures approved by auditors for disaster recovery. There will be a separate archival of electronic data performed at the end of the study to safeguard the data in accordance with regulatory requirements.

10. TRIAL MONITORING AND QUALITY ASSURANCE

10.1. Trial Monitoring Plan

The HHTU trial team will oversee trial monitoring activities, ensuring the trial is conducted in accordance with agreed SOPs, to ensure compliance with the International Conference on Harmonisation of Good Clinical Practice (ICH GCP) guidelines, as applicable under the UK Policy Framework for Health and Social Care Research. All trial related documents will be made available upon request for monitoring by HHTU monitors.

A Trial Monitoring Plan will be developed and agreed by the CI and Sponsor based on the trial risk assessment. This will include central monitoring, and both remote and on-site monitoring if required.

10.2. Trial Oversight Committees

10.2.1. Trial Management Group

The Trial Management Group (TMG) is comprised of the CI, other lead investigators (clinical and non-clinical), members with a specific interest (e.g. research assistants, PPIE representatives), members of the HHTU team and a Sponsor representative. The TMG will be held monthly for the first 2 months of the study and thereafter every 3 months of the study across the full 20 months. The TMG will be responsible for ensuring the study runs to the protocol.

10.2.2. Trial Steering Committee

The Trial Steering Committee (TSC) will provide overall supervision for the trial, in particular in relation to the progress of the trial, adherence to the protocol, participant safety and the consideration of any new relevant information. The TSC provides advice through its independent Chair, and is responsible for making executive decisions about the continuation of the trial. In addition, the TSC will include an independent statistician, as well as other independent expert members with clinical or other expertise relevant to the project, at least 1 PPIE representative, and the CI as a non-voting member. In addition, observers from the TMG (e.g. trial manager, trial statistician), Funder and Sponsor can be invited to attend at the discretion of the Chair. The TSC will meet every 6 months throughout the study.

11. SAFETY REPORTING

The collection and reporting of data on adverse events and serious adverse events will be in accordance with ICH GCP and the UK Policy Framework for Health and Social Care Research. Monitoring and reporting of safety events will be performed in accordance with HHTU SOPs and detailed in the Trial Monitoring Plan.

11.1. Safety Reporting Definitions

TERM	DEFINITION
**Adverse Event (AE)**	Any unexpected effect or untoward clinical event affecting the participant (i.e. any unfavourable and unintended sign, symptoms or disease).
**Serious Adverse Event (SAE)**	A serious adverse event is any untoward medical occurrence that:

	• results in death<br>• is life-threatening<br>• requires inpatient hospitalisation or prolongation of existing hospitalisation<br>• results in persistent or significant disability or incapacity<br>• consists of a congenital anomaly or birth defect; or<br>• is otherwise considered medically significant by the investigator.<br><br>NOTE: the term "life-threatening" in the definition of "serious" refers to an event in which the participant was at risk of death at the time of the event; it does not refer to an event which hypothetically might have caused death if it were more severe.
**Related Unexpected Serious Adverse Event (RUSAE)**	A serious adverse event which, in the opinion of the CI, resulted from administration of any of the research procedures, and is not listed in the protocol as an expected occurrence.

Note: Medical and scientific judgement must be exercised in deciding whether an event is serious. The event characteristics / consequences must be considered at the time of the event. Do not refer to an event which may hypothetically have caused one of the above."

NOTE: to avoid confusion or misunderstanding of the differences between the terms "serious" and "severe", the following note of clarification is provided: "Severe" is often used to describe intensity of a specific event, which may be of relatively minor medical significance. "Seriousness" is the regulatory definition supplied above and relates to the consequence of the event.

11.2. Adverse Event Reporting

The collection and reporting of data on adverse events (AEs) and serious adverse events (SAEs) will be in accordance with ICH GCP and the UK Policy Framework for Health and Social Care Research. Monitoring and reporting of safety aspects of the trial will be performed in accordance with HHTU SOPs and detailed in the Trial Monitoring Plan.

The AE reporting period for this study begins as soon as participants have consented to the study and ends 30 days after completion of the follow-up outcome measures for each participant. All AEs (serious and non-serious) considered by the CI to be related to the intervention will be documented at site using the AE Log and reported to HHTU via the RCC database as soon as possible after the research team is made aware of the event. A copy of the AE report and log will also be sent to the Sponsor. Any non-serious AEs that are considered not to be related to the intervention are not required to be reported to HHTU but should still be recorded in the participant's medical records if appropriate.

The CI or delegated unblinded member of the COMIC research team will be responsible for assessing AEs in relation to seriousness and causality, and this should be documented on the AE Log and in the study database. If an AE is assessed by the CI as serious, SAEs will

be reported to HHTU within 24 hours of becoming aware of the event. All SAEs from both study arms (intervention and TAU) should be reported to HHTU.

All reported AEs will be followed up by the CONIFAS2 research team until the event has resolved or a decision has been taken for no further follow-up. If a participant withdraws from the trial, the AE reporting period will end at the point of withdrawal.

A summary of all reported AEs and SAEs will be sent to the TMG for each meeting and reviewed. Any actions resulting from any patterns identified in reported AEs or SAEs will be shared with sites and distributed to participants where needed.

An SAE occurring to a research participant should be reported to the REC where in the opinion of the CI the event was:

- Related – that is, it resulted from administration of any of the research procedures; and
- Unexpected – that is, the type of event is not listed in the protocol as an expected occurrence.

Reports of related and unexpected SAEs should be submitted within 15 days of the CI becoming aware of the event, using the SAE report form for non-CTIMPs published on the HRA website.

All RUSAEs will be reviewed by the CI and will be subject to expedited reporting to the Sponsor (dependent on Sponsor processes) and REC by HHTU on behalf of the CI within 15 days.

11.3. Adverse Events of Special Interest (AESIs)

In addition to reporting AEs assessed as being related to the intervention, the following adverse events of special interest (AESIs) will also need to be reported during the study:

- Any deterioration in mental health including incidents such as self-harm, self-injurious behaviour and reported suicidal ideation or actuation.

Parents/guardians of participants in both study arms will be encouraged to report the occurrence of any of these incidents to the Trial Coordinator at COMIC LYPFT and the site clinical team. This information will then be discussed with the CI.

11.4. Recording and reporting of SAEs and RUSAEs

All SAE and RUSAE occurring from the time of informed consent until 30 days after end of trial participation must be reported by the research team within 24 hours of first knowledge of the event. Each SAE will be identified by the Participant ID Number and a sequential SAE number, and will be documented on the SAE Log held in the ISF. SAE events will be reported directly via the RCC database, although there will be a corresponding paper form to be completed in the event that RCC is not available, which will be scanned and emailed to HHTU (hhtusafetyreporting@hyms.ac.uk).

Events should be reported using the Initial SAE Report Form within the RCC study database. HHTU will receive automatic notification of all SAEs entered into RCC. HHTU will be responsible for processing safety reports for the site, and will inform the CI, second clinical assessor and Sponsor via email as soon as they become aware of the event and within 24 hours of the initial notification. If the Initial SAE Report does not include a causality

assessment, HHTU will request a Follow-Up SAE Report to be completed as soon as possible until causality has been determined.

Following query resolution, the HHTU trial management team will then release the form in sequence for the required assessments, first to the CI to assess severity, seriousness and causality, followed by assessment of expectedness by a second clinical assessor, and then a final review by Sponsor. The CI, second clinical assessor and Sponsor will sign to confirm their assessments via RCC following receipt of a notification from HHTU that their review of the event is required.

Events will be followed up until resolved or a final outcome has been reached. Follow-Up SAE Forms should be used to provide ongoing updates about the event. Several Follow-Up forms may need to be completed until the final outcome is determined by the CI. Data contained on a paper Initial SAE Report Form, only to be used if RCC is unavailable, will be entered into RCC as soon as it becomes available. There will be no paper Follow-Up SAE Report Forms, as these details will be entered directly into RCC.

For all SAEs, the following information will be collected:

- Full details in medical terms and case description
- Event duration (start and end dates, if applicable)
- Action taken
- Outcome
- Seriousness criteria
- Causality in opinion of investigator
- Whether the event would be considered expected or unexpected

11.5. Responsibilities for Safety Reporting

Chief Investigator:

- Clinical oversight of the safety of trial participants.
- Use medical judgment in assigning seriousness, causality and expectedness.
- Ensure all SAES (including RUSAEs) are recorded and reported to the HHTU within 24 hours of becoming aware and to provide further follow-up information as soon as possible.
- Ensure AEs are recorded and reported to HHTU in line with the requirements of the protocol.

Second clinical assessor:

- Use medical judgement in assigning seriousness, causality and expectedness of SAE.
- Review all Related / Unexpected events in the opinion of the CI as per protocol. In the event of disagreement between CI and second clinical assessment, CI assessment may be upgraded or downgraded by the second clinical assessor prior to reporting to the main REC.

Study sponsor:

- Maintain overall oversight of safety reporting.
- Assess causality and expectedness of SAEs.

HHTU:

- Expedited reporting of Related / Unexpected SAEs to the main REC and Sponsor (dependent on Sponsor processes) within required timelines.

- Preparing periodic safety reports to TSC.
- Notifying Investigators of Related / Unexpected SAEs which compromise participant safety.

Trial Steering Committee (TSC):

- Periodically review safety data.
- Make recommendation on the continuation of the trial based on SAEs.

12. STATISTICAL ANALYSIS

Statistical analysis is the responsibility of the UoH Trial Statistician. A full Statistical Analysis Plan (SAP) will be written and approved before any analyses are undertaken.

12.1. Sample Size

The proposed sample size is 84 (42 per arm). As a feasibility trial, the study is not powered to formally detect the effectiveness of the CONIFAS intervention versus treatment as usual (TAU). The sample size was calculated to ensure reliable estimation of key feasibility figures, detection of signal of efficacy and to provide data to inform the sample size calculation for the future full trial. Teare et al. 2014 recommend at least 35 evaluable participants per arm for external pilot / feasibility studies to robustly estimate the pooled standard deviation for a continuous outcome (22). The sample size of 84 allows for 35 participants per arm plus a 15% attrition rate.

12.2. Planned Recruitment Rate

Planned recruitment has been discussed and confirmed with the recruiting site clinical teams. It is proposed that recruitment will take 9 months, aiming for between 9 and 10 participants per month.

12.3. Statistical Analysis

The trial, including the flow of individual participants through each stage, will be reported in accordance with the CONSORT 2010 statement extension to pilot and feasibility trials (23)). A CONSORT flow diagram will be used to display patient recruitment and data completeness throughput from eligibility screening, recruitment, baseline and final follow-up visit.

Due to the nature of this feasibility study, no formal statistical tests will be undertaken. Descriptive statistics, i.e. mean (standard deviation) for continuous outcomes, medians (interquartile range, IQR) for ordinal data and raw count (%) for categorical outcomes, will be mainly employed.

At baseline, a table showing baseline demographic and clinical characteristics for each group will be presented to indicate any between group differences.

For the primary outcomes, the feasibility criteria will be recruitment rate, eligibility to consent ratio, retention rate, data quality and integrity, acceptability of the proposed outcomes and intervention fidelity. The recruitment rate, consisting of the eligibility and consent rate will be calculated with 95 % confidence intervals (CI). Feasibility outcomes will be presented in a table with reference to performance against traffic light / stop-go criteria (section 2).

The candidate primary outcome for the definitive trial, CHU9D, will be summarised by allocated group at baseline and 6-week follow-up. Also, at 6-week follow-up, the effect size with 95% CI will be calculated to assess signal of efficacy. Study secondary outcome

measures, such as SNAP-IV, RCADS and NCI, will be reported via descriptive statistics by allocated group at each time point.

Safety data (AEs and SAEs) will be summarised for each group using descriptive statistics.

13. ECONOMIC ANALYSIS

This feasibility study does not encompass a formal health economic evaluation, but the value of examining family-level impacts is recognised. To address this, questions will be asked as part of the qualitative interviews (See section 14.3), aiming to capture preliminary insights into family dynamics and resource use during the intervention. The health economist will also review the information that is gathered in the daily diaries regarding activities in order to inform the potential for impacts on household finances. These insights will shape the framework for a subsequent RCT, which will employ additional validated tools that have already proven feasible to collect in similar settings, such as EQ-5D for parents / guardians and CHU9D for siblings, to systematically assess broader family impacts.

The study will also examine how feasible it is to measure impacts on clinician time to inform the cost of intervention delivery, and to measure impacts on child wellbeing within this study target population. The CHU9D (9) will be used to provide utility values allowing quality-adjusted life years (QALYs) to be calculated. A questionnaire will be developed for the clinicians delivering the introduction to the intervention and the support calls in order to test the feasibility of measuring the impact of the intervention on how they spend their time, and the overall amount of time spent delivering the CONIFAS intervention. Costs of intervention materials will be calculated and reported.

While the study does not collect quality of life impacts on parents/guardians using an instrument such as EQ5D, there is likely sufficient existing evidence to support feasibility of using EQ5D in a future RCT.

14. PROCESS EVALUATION

The process evaluation is informed by existing intervention development frameworks (15, 24, 25) and draws on mixed methods to examine the feasibility and acceptability of the intervention, enhance understanding of the underpinning mechanisms of action and examine trial processes to ensure these support recruitment and retention to the trial.

14.1. Identification of intervention mechanisms of action

A data collection form will be developed to document the proposed active ingredients of the intervention. The following information will be identified: the main intervention delivery system (e.g. support telephone calls) and the proposed theory-linked behaviour change techniques (BCTs) identified from the BCT Taxonomy v1 (26).

BCTs will be extracted into a standardised form by one researcher and 10% verified by a second researcher. Where discrepancies are identified these will be discussed and resolved producing a BCT identification protocol. A review of initial coding will be undertaken by the primary researcher following the amended BCT protocol. The updated review will be subject to a second round of 10% coding by the second researcher. This will continue until no discrepancies are identified. BCTs will only be coded when they are identified with no special coding rules for identification included.

Using The Theory and Technique Tool (27) all identified BCTs will be mapped to a proposed mechanism of action. Only those identified as a “link” will be documented. Where no mechanism of action is identified in the online tool, mapping of BCTs to the Theoretical Domains Framework will be reviewed to identify a proposed mechanism of action.

Identified content and mechanisms of action will be used to further refine the logic model produced as part of CONIFAS1 (21), articulating the intervention programme theory. This will be used to support development of questions to ask during interview to explore the proposed BCTs as well as the mechanisms of action. It will also present an opportunity to collect data and explore mediators of intervention in subsequent work.

14.2. Patterns of implementation and fidelity

To understand whether the intervention can be delivered as planned (with fidelity) a bespoke fidelity measure has been developed and will be completed to detail the introductory session content delivered, and content of fortnightly check-in calls. Clinicians will be provided with a topic guide for supporting the calls to participants to guide their calls. This is based on the calls undertaken during CONIFAS1 (NIHR203043). Staff will be asked about their experiences rolling out the intervention in practice (implementation) as part of the qualitative interviews.

To gain an understanding of the implementation and fidelity of the intervention, a simple daily diary sheet will be completed by participants. The diaries ask individuals to document the frequency (how often) and duration (time) spent engaging in CONIFAS activity, and the activity or activities completed. This will be supplemented by the interviews. The diary format will be kept simple as user feedback from the first CONIFAS study indicated that the longer reflective diary used was not completed frequently by parents/guardians.

14.3. Semi-structured interviews: Acceptability, context and mechanisms of action

Semi-structured qualitative interviews will be undertaken with family dyads who have received the intervention from each site (parents/guardians and/or their child/ren), and families recruited to the control arm (n=12-15), after they have completed their six-week participation in the main study. Recruitment for the interviews will be staggered to the main study recruitment start to account for the six-week trial intervention period. The length of interviews will be between 30-60 minutes. Purposive sampling will focus on collecting data from those who complete the intervention, and those that withdraw. Interviews will be conducted in person (at an agreed location) online or over the telephone, depending on the preference of the interviewee. A small sample of older children will be interviewed alone, with parental agreement. Interviews will be undertaken by researchers with experience of working with this age group.

Online/phone interviews with staff (n=4-5) will explore delivery of the intervention and study, as well as discuss implementation issues. A convenience sample of staff involved in study delivery will be recruited.

14.4. Recruitment and consent

All details on parent/child dyads or older children who consented to be contacted for interviews will be collated and a diverse sample from those who consented will be purposively selected in order to collect data from those who complete the intervention, and those that withdraw, whilst also seeking to ensure diversity in site, age, gender, and co-

morbid diagnoses. Interviews will be conducted in person (at an agreed location) or online, depending on the preference of the interviewee.

Participants will be contacted by phone or email to arrange the interview at the end of their involvement in the study. Participants will be reminded of the purpose of the interviews (as outlined in the initial PIS) and provided with an interview-specific PIS. They will be offered the opportunity to ask any questions. An interview date and time will be selected to suit all parties. A sample size of 12-15 participant dyads (parent/child) is proposed, however interviews will continue to be conducted until theme saturation is achieved.

Staff participating in delivery of the intervention will be directly approached by the research team and asked if they wish to participate, ensuring purposive selection of staff to represent site, band and professional experience.

Consent process

Parents/guardians and professionals interested in participating in an interview will be provided with an interview-specific PIS. As with the consent process for the main trial, they will be able to provide informed consent via eConsent in Box Sign, via remote witnessed consent or, if requested, a member of the CONIFAS2 research team will provide a face-to-face visit to complete eConsent (see section 4.3 for details). Consent will then be verbally re-confirmed at the start of the interview. Following the interviews, each participant will be offered a voucher of £25 as a thank you.

14.5. Interview Schedule

Topic guides will be used to focus the interviews with separate topic guides for parents/guardians, older children and healthcare professionals. However, as the interviews are semi-structured the exact questions will be responsive to the individuals. Questioning will follow "funnelling" principles, where interviews are broad and less structured before becoming focused (28). A visual prompt has been created to show participants at the start of the interview and a nature activity box may also be utilised to provide visual prompts for the interview questions. Where parents have taken photos of activities, these can also be used to prompt discussion.

Interviews will be conducted by the COMIC researchers. All interviews will be audio- or video-recorded, with consent, using an encrypted dictaphone or MS Teams and transcribed verbatim. It is anticipated that interviews will last around 30-60 minutes. Data will be stored securely in line with data management policies.

Whilst initial topic guides have been developed for Health Research Authority (HRA) approvals, the guides will be shared and further developed in consultation with our PPIE and advisory groups with separate guides tailored to intervention recipients (parent/child dyad) and providers (NHS staff).

Broadly, the topics covered will include the following, however they are tailored to the audience:

- Perspectives on the proposed mechanisms of action informed by the behaviour change technique taxonomy V1 (26), Theoretical Domains Framework (17) and the Online Theory and Technique Tool (29).
- Experiences of any barriers or enablers to participation/engagement or delivery

- Implementation of intervention as both a parent/child participant as well as within the NHS care pathway

14.6. Qualitative Analysis

Data will be analysed using Framework analysis (30), which will allow for both *a priori* issues to be explored as well as the creation of data driven themes. The analysis will follow the five steps of Framework Analysis outlined below.

1. Familiarisation

Reading each transcript, whilst listening to the audio-recording to ensure any identifiable data is redacted will support familiarisation. Each participant will be referred to by a pseudonym to maintain confidentiality.

2. Identification of a framework

Where theory has been used *a priori*, these will be used for analysis. The Theoretical Domains Framework (17), and the Online Theory and Technique Tool (29) will be used to code the mechanisms of action. The Theoretical Framework of Acceptability (31) will be used to explore acceptability of the intervention. Barriers and enablers to participation will be analysed using the Theoretical Domains Frameworks (17) offering the ability to identify strategies to support implementation in further research.

An analytical framework will be developed for analysis of questions not underpinned by theory (i.e. those on impact and processes). The first 3 transcripts will be coded. These codes will then be grouped and labelled to produce an analytical framework. This framework will be subject to revisions until no new codes emerge.

3. Indexing

Data will be managed using an appropriate electronic system such as NVivo or Microsoft Excel. The appropriate framework(s) will be systematically applied to each transcript and the data units categorised into the framework categories. Where data does not fit into the framework, it will be coded to an ‘other’ category. Data will be placed into the relevant category. Where data may fit more than one category it will be coded into all those that are relevant.

4. Charting

The indexed data arranged in the framework categories will be organised and summarised into charts, with each row representing a framework category and each column a participant.

5. Mapping and Interpretation

The final stage will focus on pulling together key characteristics of the data and interpreting as a whole. This will focus on identifying patterns across the data and articulating sense-making of the data. These will be articulated in themes. In particular the focus will be on answering the research questions on the acceptability and feasibility of delivering the CONIFAS intervention with solution-focussed outcomes to guide subsequent work. In addition it will look to further refine the programme theory.

At steps 2-5 the research team will examine the ongoing analysis to ensure consistency, and transparency of process.

15. ETHICAL AND REGULATORY CONSIDERATIONS

15.1. Regulatory Compliance

The trial will be performed subject to Research Ethics Committee (REC) favourable opinion, Health Research Authority (HRA) approval, and Sponsor and participating site R&D permissions. The trial will be conducted in accordance with the principles of Good Clinical Practice (GCP) in clinical trials, as applicable under UK regulations, the UK Policy Framework for Health and Social Care, and through adherence to HHTU Standard Operating Procedures (SOPs).

Before a site can enrol participants in the study, a Capacity and Capability (C&C) assessment must be completed and confirmed by the site R&D department.

15.2. Research Ethics Committee and Health Research Authority Approval

Before the trial can open to recruitment, approval will be sought from a Research Ethics Committee (REC) and the Health Research Authority (HRA) for the trial protocol, informed consent forms, participant information leaflets, and other relevant trial documents and materials. All correspondence with the REC and HRA will be retained in the eTMF / ISF.

15.3. Amendments

All study amendments will be approved by the CI, and all substantial amendments will be approved by the CI, Sponsor, and the TSC prior to submission to the REC. The Amendment Tool should be completed for all amendments to the study and the appropriate review bodies should be notified dependent on the resulting categorisation of the amendment.

Substantial amendments that require review by the REC will not be implemented until the REC grants a favourable opinion for the trial. For any amendment that will potentially affect site activities, HHTU will request confirmation of continuing C&C from the site R&D department prior to implementing the amendment at site.

15.4. Non-Compliance

Protocol and GCP non-compliances are defined as departures from the approved protocol and/or principles of GCP identified during study implementation, data management checks and/or monitoring visits. Such non-compliances are further categorised according to the following criteria:

- Deviation: a planned or unplanned departure from the protocol or the conditions or principles of GCP that does not increase risk or decrease benefit; or have a significant impact on participant rights, safety or welfare, and/or on data integrity.
- Violation: an unplanned departure from the protocol or the conditions or principles of GCP that does increase risk or decrease benefit; or may have an impact on participant rights, safety or welfare, and/or on data integrity.
- Serious breach: a breach of the protocol or of the conditions or principles of GCP which is likely to affect to a significant degree either the safety or physical or mental integrity of the participants of the study; or the scientific value of the trial.

Planned deviations may be permitted if they are in place to protect participant safety, rights and welfare, but they should not become standard practice, and if necessary, a protocol amendment should be considered, which is particularly relevant in a feasibility study. Planned deviations or waivers relating to eligibility criteria will not be permitted. Accidental protocol deviations can happen at any time. They will be adequately documented on the relevant forms and reported to the CI and Sponsor immediately. Deviations from the protocol which are found to frequently recur are not acceptable, will require immediate action (may

trigger additional monitoring visits and refresher training sessions), and may be classified as a violation or serious breach. Prospective or planned violations or serious breaches will not be permitted under any circumstances. Any serious breaches which occur or result from frequent deviations will be reported to the NIHR.

15.5. Serious Breaches

Investigators are required to promptly notify HHTU of a serious breach (as defined in the latest version of the National Research Ethics Service (NRES) guidance). A 'serious breach' is defined as a breach of the protocol or of the conditions or principles of GCP (or equivalent standards for the conduct of non-CTIMPs) which is likely to affect to a significant degree the safety or physical or mental integrity of the trial participants, or the scientific value of the research.

Any potential serious breaches should be promptly notified to the Sponsor by the CI and HHTU.

15.6. Ethical Considerations

The trial will be performed in accordance with the recommendations guiding physicians in biomedical research involving human subjects adopted by the 18th World Medical Assembly, Helsinki, Finland, 1964, amended at the 75th World Medical Association General Assembly, Helsinki, Finland, 2024 (The Declaration of Helsinki).

Written informed consent will be obtained from participants (or their parent / legal guardian) prior to study enrolment and randomisation. The right to decline study participation without giving a reason must be respected. The participant must remain free to withdraw at any time without giving a reason and without prejudicing their further treatment. The study will be submitted to and approved by HRA approval processes and the R&D department for each participating centre prior to entering participants into the study. HHTU will provide the main REC with a copy of the final protocol, participant information sheets, consent forms and all other relevant study documentation.

Children are a vulnerable group. An age-appropriate PIS has been developed and reviewed by our PPIE group. This will be provided to potential participants to facilitate children's understanding of the study and to enable the children to be involved in the decision-making process. Assent will be sought in children <16 years if appropriate. Children will be asked to complete two outcome measures at baseline and follow-up – the CHU-9D and NCI. It is not anticipated that these measures will cause any distress to the children completing them.

The trial database will meet standards required for handling sensitive participant data, minimising the risk of data breach. HHTU staff with access to participant data will undertake mandatory training in Data Protection, GCP and SOPs.

15.6.1. Safeguarding and debriefing procedures

Researchers employed by the COMIC team at Leeds and York Partnership NHS Foundation Trust (LYPFT) and the Research and Development team at Humber Teaching NHS Foundation Trust (HTFT) will carry out participant interaction and data collection which may, at times, involve difficult or distressing conversations. To provide debriefing opportunities, they will have access to a monthly reflective practice/supervision session facilitated by an appropriately trained clinician employed by their respective organisations. They will also have monthly 1:1 meetings with their line managers and weekly study meetings with the COMIC trial TC. Should any particularly distressing situations arise, the researchers will be able to discuss these with the study CI if they wish.

15.7. Patient and Public Involvement and Engagement (PPIE)

The existing co-production group from the CONIFAS1 study have met with parents/guardians (n=6) to refine elements of the study design, including selection of a quality of life (QoL) outcome measure. Two workshops held with children in local schools were used to refine aspects of the intervention, e.g. introducing additional activities to the activity cards and changing the hot chocolate sachet to be vegan and gluten-free, though the hot chocolate sachet has now been substituted with a mini torch based on sourcing difficulties and additional recommendations. In addition, two families worked with the study team on a 1:1 basis to gain more advice on presentation and style of psychoeducational booklets which form the basis of the training for children's NHS neurodiversity service staff members.

Extensive consultation has taken place with service providers including NHS children's neurodevelopment services, social prescribing services and voluntary ADHD support organisations (York Young Person's Assembly, ADHD Foundation, Kids Charity) to consider where to situate this intervention with the decision to focus on NHS children's neurodiversity services made in conjunction with these groups. Ongoing PPIE has taken place in conjunction with COMIC team's Young Person's Advisory Network (YPAN).

PPIE work has indicated that parents and guardians using CONIFAS would like to have the opportunity to connect with both parents and guardians and for their children to have the chance to connect with other children. The intervention will therefore include the optional use of a Facebook group which will be moderated by Kids Charity and provide a community of experience space online for parents/guardians.

A public involvement group consisting of approximately 8-10 parents and their children with ADHD diagnosis will be assembled. Previous co-production children, parents and guardians will be invited to take part as well as recruiting through Young Person's Advisory Networks we are affiliated to including COMIC and Nothing About Us Without Us. This group will meet face-to face on 8 occasions throughout the study with additional online meetings taking place with parents as required, with a celebration event at Barlow Common Nature reserve at the end of the study. Additional PPIE events will be planned throughout the study term to deliver the workstream looking at increasing equality, diversity and inclusion. PPIE workshops will be facilitated by the CI and COMIC Trial Coordinator.

The Patient Involvement in Research Impact Toolkit (PIRIT) from the NIHR Learning for Involvement platform will be used. The groups will develop iteratively with new challenges addressed as the study progresses. A variety of methods will be used to enable productive involvement including creative activities and also some online or 1:1 sessions if required. This opportunity will be advertised with existing networks and also seek to ensure wider representation by sharing with ethnically diverse community groups with established links, for example Hull Afro Caribbean society. Additionally the project will be part of the COMIC YPAN which enables wider engagement with young people's groups across the Yorkshire region. Humber Teaching NHS Foundation Trust has several additional Young Person's steering groups (Humber Youth Action Group) where updates can be presented and specific support can be sought. It is intended this approach will facilitate both a core PPIE team to support and advise on specific aspects of the study as well as seek wider viewpoints from other groups of young people.

The work undertaken with the PPIE group will be varied and a collaborative approach whereby it will be discussed with the group at the first meeting how and where they feel they wish to contribute. Ideas however for involvement include:

- Co-creation of accessible summaries of the study as it progresses in a quarterly study newsletter and across social media at more regular time points
- Decisions surrounding visual elicitation methods
- Interview schedule refinement
- Data analysis refinement in the interview study
- Identification of barriers to participation and possible solutions

PPIE will be evaluated using informal questions and a formal tool, which will be selected through public involvement work but may include Gibson's Cube or a feelings thermometer.

16. CONFIDENTIALITY

All information collected during the course of the trial will be kept strictly confidential. Information will be held securely either on paper and / or electronically at HHTU, study sites and in the case of interview data at COMIC or HTFT. A project specific DPIA will be completed before collection of any participant data commences. HHTU will comply with all aspects of GDPR 2018. Operationally this will include:

- Consent from participants to record personal details: name, date of birth, address, telephone number, NHS number, GP name and address and co-morbid diagnoses.
- Appropriate storage, restricted access and disposal arrangements for participant personal and clinical details.
- Consent from participants for access to their medical records by responsible individuals from the research staff or from regulatory authorities, where it is relevant to trial participation.
- Consent from participants for the data collected for the trial to be used to evaluate safety and develop new research.
- Fully identifiable information is logically separated from all study data.
- Identifiable data is tagged as such during database development. Identifiable data is excluded from study database downloads by default. As such there is no chance of accidental export of identifiable information.
- All HHTU systems are within scope of the HHTU NHS Data Security and Protection Toolkit. These have been independently checked by the NHS Digital Information Security Team. See https://www.dsptoolkit.nhs.uk/OrganisationSearch/EE133824-HHTU.
- Where central monitoring of source documents by HHTU (or copies of source documents) is required, the participant's name must be redacted by site before sending.
- Where anonymisation of documentation is required, the sites are responsible for ensuring only the instructed identifiers are present before sending to HHTU.

All site investigators and research staff must comply with the requirements of GDPR 2018 with regards to the collection, storage, processing and disclosure of personal information, and will uphold the Regulation's core principles.

HHTU, COMIC and the Sponsor will maintain the confidentiality of all participants' data in accordance with GDPR 2018 and will not reproduce or disclose any information by which participants could be identified. Representatives of HHTU and the Sponsor will need access to participants' clinical records for the purposes of monitoring and source data verification. Confidentiality will be maintained at all times. Representatives of regulatory authorities may require access to participants' clinical records for regulatory purposes only. These points will be made clear in the PIS and ICF.

For those who choose to join the peer support Facebook forum, participant names will be visible to others. This will be created as a closed group with agreement to a confidentiality clause mandatory to join group. The wish to join the group will be indicated as an optional statement on the ICF.

17. INDEMNITY

Humber Teaching NHS Foundation Trust will act as the main Sponsor for this study and oversee the delivery of the study with Hull Health Trials Unit and Leeds and York Partnership Foundation Trust. Humber Teaching NHS Foundation Trust and Leeds and York Partnership Foundation Trust hold standard NHS Hospital Indemnity and insurance cover with NHS Resolution for NHS Trusts in England, which apply to this study to meet the potential legal liability of the sponsor for harm to participants arising from the management of the research.

Hull Health Trials Unit holds negligent harm and non-negligent harm insurance policies which apply to this study and the involvement of Hull Health Trials Unit for harm to participants arising from the design of the research. The risk exposures under the study would fall under our combined employer's and public liability insurance cover which would respond to a claim.

18. DISSEMINATION

The research will produce a range of outputs including the physical intervention and training ready for delivery, outputs to communicate the results of the study and resources to inform a future randomised controlled trial. A range of potential audiences to be targeted with the intervention have been identified, as have specific conferences and publications to target.

NIHR: the study will produce a research protocol, and other associated documentation including a bespoke fidelity checklist for a full RCT which will include all refinements resulting from the feasibility study.

Clinicians, service providers: the research will create a physical co-produced resource for parent/guardian and child use at home (box with activities/psychoeducation) alongside an accompanying training package including videos and written materials to support Children/s Neurodiversity Service staff with the intervention delivery.

Children and families: child-friendly and accessible dissemination of the findings will be co-developed with the PPIE group. Findings will be shared widely through the COMIC YPAN with young people, schools and the general public at community events. The results of the study will inform the design of a full trial of the intervention and provide further understanding of how parents and staff interact with the intervention.

Wider stakeholders: accessible summaries and updates will be produced to share via websites (University, NHS, ADHD partners, KIDS, Wildlife Trusts) and on social media. The study team has national reach through its partner organisations and access to present at national conferences including through the local ICS who are supportive of the research. Findings will be published in scientific journals (e.g. Journal of Child and Adolescent Mental Health, BMC Paediatrics, Child Health Care, BMJ Open) and disseminated more widely through infographics, short reports and posters on affiliated websites and presentations at conferences. These outputs will be tailored to the specific audiences focusing on preparation for a full RCT. For example, outputs to engage children and their parents will be disseminated through appropriate channels to support engagement and participation in a wider RCT – creating a waiting audience.

Health economic data and acceptability and feasibility data will be used to inform reports to take to a wider strategic level for engagement for a full RCT. At a service provider level data collected on delivery acceptability and feasibility as well as addressing identified barriers, will be used in outputs such as presentations, to engage with other Children's Neurodiversity Services teams to create a list of sites for participation in a full RCT.

The study will produce a finalised version of the physical nature activity box intervention as well as a manual detailing training for interventionists and all elements of intervention delivery including training to staff through PowerPoint presentations and the intervention videos.

The findings of the study will be presented in multiple formats including accessible infographics, an animated video, and at least one academic publication (Journal of Child and Adolescent Mental Health, BMC Paediatrics, Child Health Care, BMJ Open). Further formats for study dissemination will be decided upon by PPIE in collaboration with the research team. Findings will also be presented at a CONIFAS2 dissemination event (either in-person or online) at the end of the study and at research events and conferences (to include regional Applied Research collaboration events, Integrated Care System events, NHS Community of Practices, Regional Research Delivery Network events).

The study will be adopted as part of a portfolio of work being supported by the Centre for Addiction and Mental Health Research (University of Hull). This will enable national and regional opportunities to disseminate CONIFAS2. Findings will be targeted towards those for whom they are important: families with ADHD including children, parents, carers/guardians, and wider family members and professionals working in this area (allied health professionals, charity workers, education leaders).

The findings of the study will be used to inform a larger RCT to test the efficacy of the intervention for this population. Either NIHR HTA or RfPB would be suitable funders for a definitive trial application.

18.1. Publication Policy

Prior to trial recruitment, the trial will be registered with an authorised registry, according to the International Committee of Medical Journal Editors (ICMJE) Guidelines. This is likely to be the International Standard Randomised Controlled Trial Number Register (ISRCTN) as recommended by NIHR.

The success of the trial depends on the collaboration of all participants. For this reason, credit for the main results will be given to all those who have collaborated on the trial, through authorship and contributions. Authorship guidelines will be provided for manuscripts

submitted to medical journals. These state that authorship credit should be based only on substantial contribution to:

- conception and design, or acquisition of data, or analysis and interpretation of data;
- drafting the article or revising it critically for important intellectual content; and
- final approval of the version to be published

and that all these conditions must be met (www.icmje.org).

To maintain trial scientific integrity, data will not be released before the first publication of primary endpoint analysis, either for publication or oral presentation, without TSC permission. Individual collaborators must not publish data concerning their participants before the first publication of primary endpoint analysis.

All publications must acknowledge the funding from the NIHR Research for Patient Benefit (RfPB) funding scheme and contain a disclaimer:

Funder statement:

"This research is funded by the National Institute for Health Research for Patient Benefit (NIHR RfPB) Programme (NIHR 207869)"

Disclaimer:

"The views and opinions expressed therein are those of the authors and do not necessarily reflect those of the NIHR RfPB Programme, NIHR, NHS or the Department of Health and Social Care.

Requirements in relation to placing NIHR logos on outputs varies according to output types and either the named activity coordinator should be contacted to clarify this, or the online guidance should be consulted: NIHR-Brand-Guidelines-V1.0-February-2019.pdf The logo should not be included in research reports or articles.

The CONIFAS2 Trial Steering Committee (TSC) should have access to any main study reports and results papers that may be produced and may review and comment on these papers before publication. Open Access publishing routes should be considered as the NIHR supports the principle of Open Access to research as set out in its statement supporting UKPubMed Central:
http://www.nihr.ac.uk/files/pdfs/OpenAccessPolicyStatement.pdf

19. REFERENCES

1. McKechnie DG, O'Nions E, Dunsmuir S, Petersen I. Attention-deficit hyperactivity disorder diagnoses and prescriptions in UK primary care, 2000–2018: population-based cohort study. BJPsych Open. 2023;9(4):e121.
2. Klassen AF, Miller A, Fine S. Health-related quality of life in children and adolescents who have a diagnosis of attention-deficit/hyperactivity disorder. Pediatrics. 2004;114(5):e541-e7.
3. House T. CAMHS crisis - the long wait for neuro assessments. 2023.
4. Psychiatrists RCo. Statement of shortage of ADHD medication 2023 [Available from: https://www.rcpsych.ac.uk/news-and-features/latest-news/detail/2023/10/19/statement-on-shortage-of-adhd-medication.

5. Sampaio F, Feldman I, Lavelle TA, Skokauskas N. The cost-effectiveness of treatments for attention deficit-hyperactivity disorder and autism spectrum disorder in children and adolescents: a systematic review. European child & adolescent psychiatry. 2022;31(11):1655-70.
6. DuPaul GJ, Evans SW, Mautone JA, Owens JS, Power TJ. Future directions for psychosocial interventions for children and adolescents with ADHD. Journal of Clinical Child & Adolescent Psychology. 2020;49(1):134-45.
7. de Vries S, Verheij R. Residential green space associated with the use of attention deficit hyperactivity disorder medication among Dutch children. Frontiers in Psychology. 2022;13:948942.
8. NICE. Attention deficit hyperactivity disorder: diagnosis and management: NICE guideline [NG87]. 2019.
9. Gardner B, Lally P, Wardle J. Making health habitual: the psychology of 'habit-formation'and general practice. British Journal of General Practice. 2012;62(605):664-6.
10. McEwan K, Ferguson FJ, Richardson M, Cameron R. The good things in urban nature: A thematic framework for optimising urban planning for nature connectedness. Landscape and Urban Planning. 2020;194:103687.
11. Trust N. The pandemic has driven an unprecedented surge in demand for mental health services for children and young people. 2022.
12. Fyfe-Johnson AL, Hazlehurst MF, Perrins SP, Bratman GN, Thomas R, Garrett KA, et al. Nature and children's health: a systematic review. Pediatrics. 2021;148(4).
13. Meredith GR, Rakow DA, Eldermire ER, Madsen CG, Shelley SP, Sachs NA. Minimum time dose in nature to positively impact the mental health of college-aged students, and how to measure it: A scoping review. Frontiers in psychology. 2020;10:488425.
14. White MP, Alcock I, Grellier J, Wheeler BW, Hartig T, Warber SL, et al. Spending at least 120 minutes a week in nature is associated with good health and wellbeing. Scientific reports. 2019;9(1):7730.
15. Skivington K, Matthews L, Simpson SA, Craig P, Baird J, Blazeby JM, et al. Framework for the development and evaluation of complex interventions: gap analysis, workshop and consultation-informed update. Health technology assessment (Winchester, England). 2021;25(57):1.
16. Michie S, Atkins L, West R. The behaviour change wheel. A guide to designing interventions. 2014;1:1003-10.
17. Cane J, O'Connor D, Michie S. Validation of the theoretical domains framework for use in behaviour change and implementation research. Implementation science. 2012;7:1-17.
18. Mellor K, Albury C, Dutton SJ, Eldridge S, Hopewell S. Recommendations for progression criteria during external randomised pilot trial design, conduct, analysis and reporting. Pilot and Feasibility Studies. 2023;9(1):59.
19. Furber G, Segal L. The validity of the Child Health Utility instrument (CHU9D) as a routine outcome measure for use in child and adolescent mental health services. Health and Quality of Life Outcomes. 2015;13:1-14.
20. Peasgood T, Bhardwaj A, Biggs K, Brazier JE, Coghill D, Cooper CL, et al. The impact of ADHD on the health and well-being of ADHD children and their siblings. European child & adolescent psychiatry. 2016;25:1217-31.
21. Armitt HA, Kingsley EN, Attwell L, White PC, Woolley K, Garside M, et al. Co-production of a nature-based intervention for children with ADHD study (CONIFAS): Creating a home-based intervention with children and families with lived experience of ADHD. Children and Youth Services Review. 2025;169:108042.
22. Teare MD, Dimairo M, Shephard N, Hayman A, Whitehead A, Walters SJ. Sample size requirements to estimate key design parameters from external pilot randomised controlled trials: a simulation study. Trials. 2014;15:1-13.
23. Eldridge SM, Chan CL, Campbell MJ, Bond CM, Hopewell S, Thabane L, Lancaster GA. CONSORT 2010 statement: extension to randomised pilot and feasibility trials. bmj. 2016;355.

24. Moore GF, Audrey S, Barker M, Bond L, Bonell C, Hardeman W, et al. Process evaluation of complex interventions: Medical Research Council guidance. bmj. 2015;350.
25. May C. Towards a general theory of implementation. Implementation Science. 2013;8(1):18.
26. Michie S, Richardson M, Johnston M, Abraham C, Francis J, Hardeman W, et al. The behavior change technique taxonomy (v1) of 93 hierarchically clustered techniques: building an international consensus for the reporting of behavior change interventions. Annals of behavioral medicine. 2013;46(1):81-95.
27. Johnston M, Carey RN, Connell Bohlen LE, Johnston DW, Rothman AJ, de Bruin M, et al. Development of an online tool for linking behavior change techniques and mechanisms of action based on triangulation of findings from literature synthesis and expert consensus. Translational Behavioral Medicine. 2020;11(5):1049-65.
28. Morgan DL. Focus groups as qualitative research: Sage; 1997.
29. Johnston M, Carey RN, Connell Bohlen LE, Johnston DW, Rothman AJ, De Bruin M, et al. Development of an online tool for linking behavior change techniques and mechanisms of action based on triangulation of findings from literature synthesis and expert consensus. Translational behavioral medicine. 2021;11(5):1049-65.
30. Ritchie J, Spencer L. Framework analysis. Qualitative. 1994.
31. Sekhon M, Cartwright M, Francis JJ. Acceptability of healthcare interventions: an overview of reviews and development of a theoretical framework. BMC health services research. 2017;17:1-13.
